# In Human Autoimmunity, a Substantial Component of the B Cell Repertoire Consists of Polyclonal, Barely Mutated IgG^+ve^ B Cells

**DOI:** 10.3389/fimmu.2020.00395

**Published:** 2020-03-20

**Authors:** Graeme J. M. Cowan, Katherine Miles, Lorenzo Capitani, Sophie S. B. Giguere, Hanna Johnsson, Carl Goodyear, Iain B. McInnes, Steffen Breusch, David Gray, Mohini Gray

**Affiliations:** ^1^Ashworth Laboratories, School of Biological Sciences, Institute of Immunology and Infection Research, The University of Edinburgh, Edinburgh, United Kingdom; ^2^MRC/University of Edinburgh Centre for Inflammation Research, The Queen's Medical Research Institute, Edinburgh, United Kingdom; ^3^Harvard Medical School, Boston, MA, United States; ^4^College of Medical, Veterinary and Life Sciences, Institute of Infection, Immunity and Inflammation, University of Glasgow, Glasgow, United Kingdom; ^5^Orthopaedic Unit, Royal Infirmary of Edinburgh, Edinburgh, United Kingdom

**Keywords:** B cells, BCR, autoimmunity, TNF, rheumatoid arthritis

## Abstract

B cells are critical for promoting autoimmunity and the success of B cell depletion therapy in rheumatoid arthritis (RA) confirms their importance in driving chronic inflammation. Whilst disease specific autoantibodies are useful diagnostically, our understanding of the pathogenic B cell repertoire remains unclear. Defining it would lead to novel insights and curative treatments. To address this, we have undertaken the largest study to date of over 150 RA patients, utilizing next generation sequencing (NGS) to analyze up to 200,000 BCR sequences per patient. The full-length antigen-binding variable region of the heavy chain (IgGHV) of the IgG B cell receptor (BCR) were sequenced. Surprisingly, RA patients do not express particular clonal expansions of B cells at diagnosis. Rather they express a polyclonal IgG repertoire with a significant increase in BCRs that have barely mutated away from the germline sequence. This pattern remains even after commencing disease modifying therapy. These hypomutated BCRs are expressed by TNF-alpha secreting IgG^+ve^CD27^−ve^ B cells, that are expanded in RA peripheral blood and enriched in the rheumatoid synovium. A similar B cell repertoire is expressed by patients with Sjögren's syndrome. A rate limiting step in the initiation of autoimmunity is the activation of B cells and this data reveals that a sizeable component of the human autoimmune B cell repertoire consists of polyclonal, hypomutated IgG^+ve^ B cells, that may play a critical role in driving chronic inflammation.

## Introduction

Rheumatoid arthritis (RA) is the commonest autoimmune inflammatory arthritis, affecting up to 1% of the world's population (1, 2). It is characterized by chronic systemic inflammation that targets the synovial joints, leading to progressive joint damage and disability. Whilst pathogenesis is incompletely understood, the pivotal role of B cells is supported by the efficacy of B cell depletion therapy (BCDT) in the majority of treated patients (2–4). Auto-antibodies binding to post-translationally modified proteins (e.g., ACPA) or the constant region of other immunoglobulins [rheumatoid factors (RF)] (5), augment the generation of immune complexes. However, there is a poor correlation between a good clinical response to BCDT and a reduction ACPA titer (6, 7), suggesting that other B cell specificities may be more important in driving chronic inflammation. Sjögren's syndrome is a systemic, autoimmune, chronic inflammatory disease, characterized by auto-antibodies and lymphocytic infiltration of exocrine glands. It affects about 0.1% of the population but is three times commoner in patients with RA. Currently there are no licensed treatments that affect the prognosis of Sjögren's syndrome and RA remains incurable.

The advent of accessible, high throughput sequencing technologies has enabled formal evaluation of differences between the B cell repertoire of healthy and diseased individuals. To date, studies characterizing the B cell repertoire of RA patients have been limited in number, sample size or sequencing depth (8). An appreciation of the expressed BCR repertoires in RA patients would provide a more complete understanding of disease pathogenesis. We hypothesized that RA patients would have expansions of circulating pathogenic B cells at diagnosis, that could also be detected as a conserved BCR signature in established disease. Utilizing next generation sequencing (NGS) of peripheral blood and synovial B cells, we sequenced the repertoire of expressed BCRs, focusing on the main antigen binding IgG variable heavy (IgGHV) region. We assessed 127 newly diagnosed RA patients, 16 patients with established RA and 8 paired blood and synovial samples. In addition, we phenotyped peripheral blood B cells from an additional 64 RA patients and 30 healthy controls. RA patients expressed significantly more IgG^+ve^ BCR sequences with fewer than five mutations, which we refer to hereafter as hypomutated (or IgG^hypoM^). A similar response was seen in patients with Sjögren's syndrome. The hypomutated IgGHV BCRs were polyclonal and originated from IgG^+ve^CD27^−ve^ cells that secreted significantly more TNF-alpha when stimulated, and which were significantly increased in the circulation. We also detected minimal sharing of identical IgGHV BCRs either between patients or between the synovium and peripheral blood of the same patient. RA patients also expressed significantly more IgG^+ve^CD27^−ve^ B cells that lacked expression of CD24, CD38, and CD21, which are akin to double negative 2 (DN2) (9) B cells known to be associated with more severe, active systemic lupus erythematosus (SLE) (10, 11). The prevalence of IgG^hypoM^ expressing B cells leads us to hypothesize that they may play a key role in driving chronic inflammation in systemic autoimmunity.

## Materials and Methods

### Ethical Review and Donor Selection Criteria

The use of human samples for cohorts 1, 3, and 4 was approved by the South East Scotland Bioresource NHS Ethical Review Board (Ref. 15/ES/0094). Ethical permission to collect samples donated to the SERA inception (cohort 2) was approved by the West of Scotland Local Research Ethics Committee (Ref. 10/S0703/4) as previously described (12). Informed consent was obtained from all study participants prior to sample collection. Patient cohorts are described in Supplementary Tables 1–4.

### Flow Cytometry & FACS Sorting

PBMC were stained in PBS with 1% FCS for 20 min at 4°C. A BD Aria II was used for flow sorting and a BD LSRII was used to collect data. All analysis was performed using FlowJo software. Debris and dead cells were excluded using FSC-SSC. Doublets were excluded using both FSC and SSC singlet gating. The full gating strategy is shown in Supplementary Figures 8, 9. A list of antibody reagents is shown in Supplementary Table 6. Intracellular staining was performed, following stimulation (for 4.5 h) with PMA [Sigma (20 ng/ml)] & Ionomycin [Sigma (1 μg/ml)]. After 1 h of stimulation Brefeldin A [Sigma (1 μg/ml)] was added for the remaining 3.5 h. Surface staining was performed before fixation and permeabilization using a Cytofix/Cytoperm kit (BD Biosciences).

### Cell Purification

Peripheral blood mononuclear cells (PBMC) were prepared from citrated blood samples using Ficoll® Paque Plus density centrifugation following manufacturer's instructions (GE Healthcare). Synovial tissue was dissected followed by digestion for 2 h at 37°C in 1 mg ml^−1^ Collagenase 1 (Sigma-Aldrich). Debris was removed and cells isolated by sequentially passing through 100, 70 and 40 μm cell strainers (Corning). PBMC and synovial tissue were enriched for B-cells using either anti-CD19 magnetic beads or anti-CD20 (Miltenyi Biotech) as outlined in Supplementary Table 5.

### B Cell Repertoire Sequencing

B cell repertoire sequencing was performed as previously described (13, 14), with the modifications described below. Supplementary Table 5 specifies the individual amplification strategies employed for samples from each cohort where variations were present. mRNA was purified using mRNA direct kit (Life Technologies), or total RNA was purified using either a Direct-zol total RNA kit (Zymo Research) or a Paxgene blood RNA kit (Qiagen). First strand cDNA synthesis was performed using Superscript III first-strand synthesis supermix (Invitrogen) or total cDNA synthesis kit (Applied Biosystems) according to the manufacturer's recommended protocols. For samples where low cell counts were included (CD27 sorted B-cells and synovial cells, as indicated in Supplementary Table 5), whole transcriptome amplification was performed using Smartscribe Reverse Transcriptase and Advantage2 PCR kits (Takara BioEurope) following the Smart-Seq2 protocol (15). For all samples, V-region amplicons were generated by PCR using Phusion Flash polymerase (ThermoFisher Scientific) with individual pools of forward primers within framework region 1 (FR1) designed to amplify all known V-region alleles, and a reverse primer within the IgG or IgM constant regions (Supplementary Figure 1). Samples were pooled and run on a 2% agarose gel and the dominant band was purified from an excised gel band equivalent to 400–450 bp. 250 bp paired-end sequencing of the pool of PCR amplicons was performed on an Illumina MiSeq sequencer using MiSeq Version 2 500 cycle kit with a pool of read 1 sequencing primers, an indexing primer, and read 2 constant region amplification primers. Read 1 and read 2 sequencing primer sequences were identical to the pool of amplification primers detailed in Supplementary Figure 1 but omitting the Illumina adaptor sequence. All sequence data generated in this work has been deposited in the NCBI Short Read Archive under project number PRJNA561156.

### Immune Repertoire Analysis

Sequence read-pairs were trimmed to remove sequence below a quality threshold of Q30 then combined into sequence contigs using the FLASH utility version 1.2.11 (16), and sequences with an overlap of fewer than 15 bp, or mismatch ratio of ≥0.25 were excluded from further processing. Sequences were aligned to the human germline V, D, and J segment alleles from the IMGT database using the VDJfasta utility (17), with all sequences that were successfully aligned to human IGMT V alleles being taken forward for further analysis. Donors were excluded from the study if the repertoire contained fewer than 25,000 total reads after processing with VDJfasta as this was indicative of poor sample preservation or preparation, and any such donors are not shown in the patient information tables for this study. Mutation counts and frequencies were generated from the VDJfasta utility, as the number of nucleotide differences between the sequencing read of each donor and the predicted germline Vh segment allele, allowing the number of Vh segment mutations to be determined. Frequency distributions for each donor were derived using the ggplot2 (18) and plyr (19) packages of the R statistical package (20). Skewness is a measure of the asymmetry of the distribution about its mean, and was calculated as the adjusted Fisher-Pearson standardized moment coefficient (21) using the skew method of the Pandas Python library (22).

Clonal abundance analyses were performed by grouping all reads with identical CDR3 amino acid sequences, where the predicted germline Vh allele was identical. This clone was assigned the mean number of hypermutations for all reads contributing to the clone. Clonal clustering analyses, as shown in Supplementary Figures 10, 11, were performed by first grouping sequences by shared V gene, J gene, and junction length (23). Clonal clusters then were generated by single-linkage clustering of CDR3 sequences within a Hamming distance of 1. Repertoire clonal overlap scores were used to assess repertoire similarity (24), and were here calculated as the total number of reads from shared clonal clusters between two samples, divided by the sum of sequencing reads in both samples, with potential values ranging from 0 to 1.

The Gini index was calculated for each sample based upon the read counts for each unique complementarity determining region 3 (CDR3) amino acid sequence in the repertoire, according to the formula:

$$\begin{array}{l} Gini\;index=\;\frac{2\sum_{i}^{n}i\times y_{i}}{n\sum_{i}^{n}y_{i}}-\frac{n+1}{n} \end{array}$$

Analysis of the paired blood/synovial B cell data from study 4 was performed by combining read pairs and prediction of germline sequences pRESTO and Change-O, followed by processing using the Alakazam package of the Immcantation adaptive immune repertoire analysis framework (23, 25). No read count threshold was applied to this data, but instead sequences were filtered using the CollapseSeq tool to include only sequences with a duplication count of 2 or greater.

### B Cell Clone Lineage Tree Construction

Multi-compartmental clones (i.e., B cell clones which were found in both peripheral and synovial B cells) were identified as B cell clones containing at least 1 sequence present in each of the paired peripheral blood and synovial samples from an individual donor. Lineage trees of multi-compartmental clones were inferred using PHYLIP v3.6 in Alakazam. The generated lineage trees were exported from R as. GML files and graph layout was plotted in Gephi v0.9.2. Nodes were colored according to sequence mutation count and scaled according to the duplication count of each sequence. Germline and inferred sequences were colored white and set to the minimum node size. Graph layout was calculated using the ForceAtlas 2 algorithm. The lineage trees of B cell clones containing sequences that displayed evidence of index misassignment were discarded.

### Statistical Analysis and Data Visualization

Before performing inferential statistical tests, data were assessed for conformity to the assumptions of the test used. The assumption of normality of data was visually assessed using the Q-Q plot method, generated using the StatsModels Python package. Prism 6.0 (GraphPad Software Inc.) or the scipy.stats Python package (26) was used to perform all Student's *T*-tests, Mann-Whitney, Wilcoxon and Kruskal-Wallis non-parametric tests. Two-tailed *p*-values are given in all cases. All plots were drawn with Prism (Graphpad Software Inc.) or with the Matplotlib (27) or Seaborn Python packages, and data processing used the Pandas package (22). Dunn's *post-hoc* test was run using the scikit-posthocs module (28). For analyses involving multiple pairwise comparisons, *p*-value adjustment was performed using the Holm-Šídák method (29). Use of the ± following mean values indicates the 95% confidence interval.

## Results

### RA Patients Express a Higher Frequency of Hypomutated IgG B Cell Receptors Within Peripheral Blood

**Figure 1:**
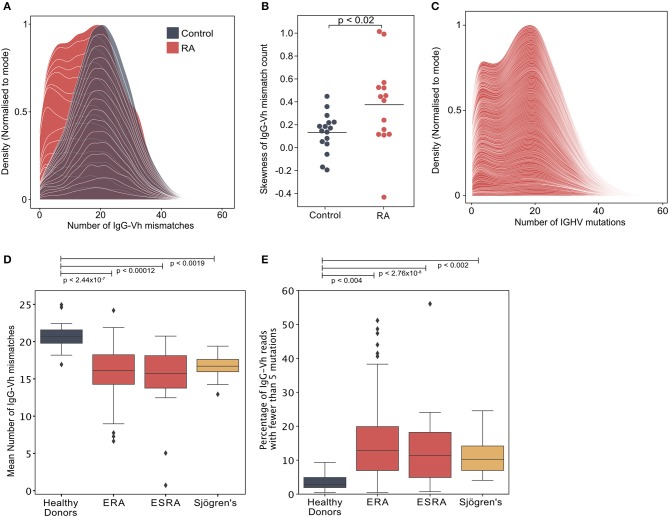
**(A)** Distribution of the number of IgG-Vh mismatches per sequencing read for DMARD naïve early RA patients (ERA) (*n* = 14) and healthy control donors (*n* = 16). Individual density plots are stacked to indicate the overall distribution across all samples in each group. Maximum cumulative density values for each group are normalized to the mode to facilitate inter-group comparison. **(B)** Skewness of IgG mutation distributions from RA patients (*n* = 14) and healthy control groups (*n* = 16). Horizontal lines denote the arithmetic mean skewness for each group. *P*-value shown was calculated using Mann-Whitney *U*-test. **(C)** Distribution of the number of V segment mismatches per sequencing read for ERA patients [cohort 2, *n* = 113]. Individual density plots are stacked to indicate the overall distribution across all samples in each group. **(D)** Mean IgG-Vh mismatches for control donors (*n* = 16), ERA donors from cohorts 1 and 2 (*n* = 14 and *n* = 113, respectively), ESRA donors from cohort 3 (*n* = 16), and Sjögren's syndrome patients (*n* = 15). *P*-values are generated by Kruskal-Wallis test with Dunn's post-test to compare the means for each RA group with the control donor group. **(E)** Percentage of IgG reads with fewer than 5 mutations for control donors (*n* = 16), ERA donors from cohorts 1 and 2 (*n* = 14 and *n* = 113, respectively), ESRA donors from cohort 3 (*n* = 16), and Sjögren's syndrome patients (*n* = 15). *P*-values are generated by Kruskal-Wallis test with Dunn's post-test to compare the median values for each RA group with the control donor group.

200,000 highly purified CD19^+ve^ B cells isolated from the peripheral blood of each of 14 newly diagnosed treatment-naïve seropositive early rheumatoid arthritis (ERA) patients (cohort 1/Supplementary Table 1) and 16 healthy controls were sequenced by NGS. The degree of mutation within the IgG heavy chain (IgGHV) was calculated by assessing the number of nucleotide mismatches between each sequence read and the closest predicted germline V segment sequence. Whereas, the number of mutations per IgG read approximated to a symmetrical distribution in healthy individuals, the distribution of mutations in RA donors was skewed by the presence of an increased frequency of poorly mutated IgG sequences (Figures 1A,B; Supplementary Figure 2). This observation was confirmed in a larger cohort of 113 newly diagnosed, disease modifying anti-rheumatic drug (DMARD) naïve, seropositive ERA patients, selected from the Scottish Early Rheumatoid Arthritis (SERA) inception cohort (12) (cohort 2/Supplementary Table 2). A bimodal distribution of IgGHV mutation counts was observed in the IgGHV sequences with the first peak representing poorly mutated IgG sequences (Figure 1C). To establish if this population persisted in patients with established RA (ESRA), we sequenced the IgGHV sequences from 16 ESRA donors (cohort 3/Supplementary Table 3A), and to ascertain if IgG^hypoM^ are a unique feature of RA, we also sequenced the peripheral blood IgG-Vh repertoires of 15 patients with primary Sjögren's syndrome (Supplementary Table 3B).

Analysis of the mean IgG mutation count per read showed that there were fewer IgGHV mutations in the ERA, ESRA and Sjögren's syndrome cohorts, compared to healthy control donors (Figure 1D). A further 12 paired samples taken 6 months following DMARD therapy (Supplementary Table 2) confirmed that the mean number of IgGHV mutations was only slightly increased [16.3 ± 2.4 compared with 12.8 ± 1.9 at diagnosis] (p < 0.006, Wilcoxon signed-rank test) (Supplementary Figure 2C). The skewed distribution of IgGHV mutation counts in ERA donors were the result of an increased frequency of IgG sequences with fewer than 5 mutations. Indeed, the mean percentage of the IgG repertoire composed of fewer than 5 V-segment mutations [hypomutated IgG sequences [IgG^hypoM^]] in the RA cohorts was significantly higher than healthy controls (means with 95% confidence intervals were 12.6% ± 1.5 ERA patients from cohorts 1 and 2, 8.4% ± 4.7 for ESRA, and 8.3% ± 2.2 for Sjögren's patients, compared to the mean for healthy controls of just 2.8% ± 0.9) (Figure 1E). The analysis of individual cohorts is shown in Supplementary Figure 2D. In contrast, there was no difference in the mean number of mutations for IgM between ERA and healthy control populations, possibly due to the inherently lower hypermutation frequency of this isotype (Supplementary Figure 3). The frequency of IgG^hypoM^ and the disease activity 28 joint score (DAS28) were not correlated, making it unlikely that the presence of IgG^hypoM^ was simply a result of more marked inflammation (Supplementary Figure 2B). Nonetheless, the existence of this hypomutated IgG population in both RA and Sjögren's syndrome patients suggests it is a generalized feature of failed B cell tolerance, contributing to human autoimmunity.

### Hypomutated Sequences Are Distributed Throughout the IgG Repertoire in RA

**Figure 2:**
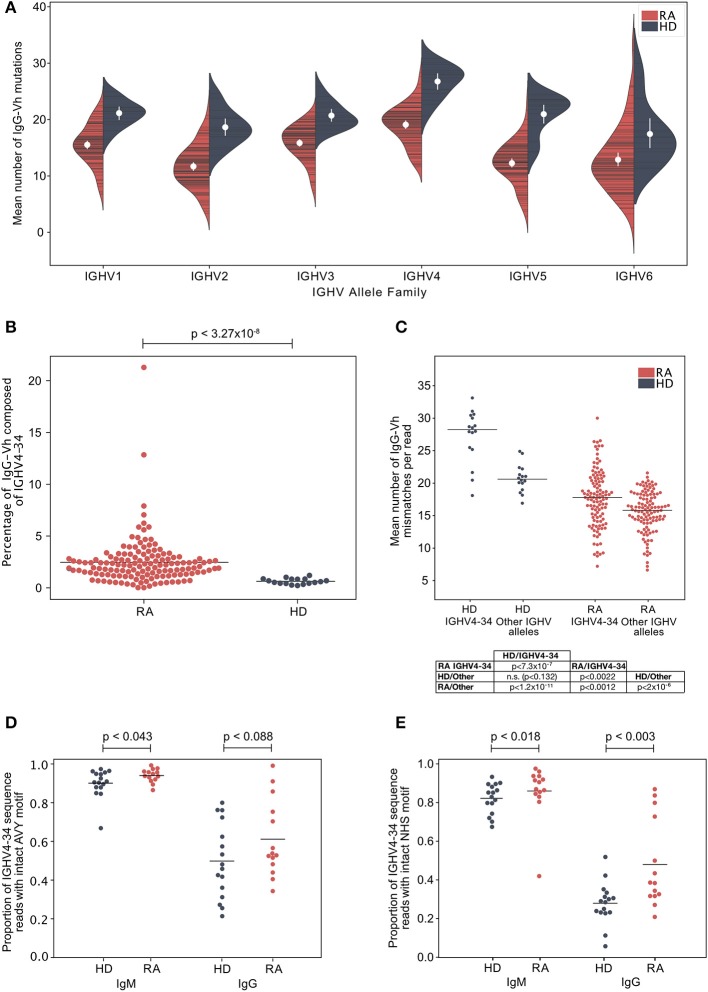
**(A)** The mean number of IgG-Vh V segment mismatches per read for each individual in the ERA (cohort 2, *n* = 113) and healthy control groups (cohort 1, *n* = 16). Data are split by germline IGHV family group. White circles denote group means, vertical white lines show the 95% confidence interval for the mean. **(B)** Percentage of IgG reads that use the IGHV4-34 allele in ERA patients (cohort 2, *n* = 113) and control donors (cohort 1, *n* = 16). Horizontal bars denote group means, and *p*-values calculated by Mann Whitney *U*-test. **(C)** Mean number of IgG-Vh mismatches per read for ERA donors (*n* = 113, cohort 2) and healthy control donors (*n* = 16). For each donor, the mean number of mutations for all reads mapping to IGHV4-34, or to other IGHV alleles, were calculated and plotted independently, with horizontal bars plotted to indicate the group mean. *P*-values calculated using Kruskal Wallis with Dunn's *post-hoc* pairwise test, and with Holm-Šídák correction for multiple comparisons of group means. **(D)** Proportion of IGHV4-34 reads of IgM and IgG isotype sequences where the carbohydrate binding AVY motif within framework region 1 (IMGT numbering 24–26) is present. *P*-value calculated using Mann-Whitney test to compare the group means. **(E)** Proportion of IGHV4-34 IgM and IgG isotype sequences where the NHS glycosylation motif within CDR2 (amino acid residues 57–59, IMGT numbering scheme) is present. *P*-value calculated using Mann-Whitney test to compare the group means.

A potential explanation for the increased frequency of IgG^hypoM^ in RA donors could be mono- or oligo-clonal expansion of IgG B cell clonotypes with few mutations. However, this was unlikely given that the IgG^hypoM^ sequences were not restricted to particular IGHV allele families (Figure 2A; Supplementary Figure 4). The IGHV4-34 gene segment, that is associated with self-reactivity and a failure of B cell tolerance and autoimmunity (30–32) is censored at multiple check points in healthy individuals (33). Yet, IGHV4-34 expression was significantly higher in the IgG^+ve^ B cells from 113 DMARD naïve RA patients (2.41%, ± 0.43) compared to healthy controls (0.65%, ± 0.16) (Figure 2B). Somatic mutations within the IGHV4-34 gene reduce self-reactivity (34, 35) but the IGHV4-34 allele in RA IgG^+ve^ BCRs expressed significantly fewer mutations compared to the healthy controls (*p* < 7.31 × 10^−7^) (Figure 2C). This demonstrates that RA patients generate considerably more IgG^+ve^ B cells that utilize a poorly mutated IGHV4-34 allele. The IGHV4-34 allele is unusual in that it contains an Ala-Val-Tyr (AVY) motif (within the framework 1 region) responsible for the self-reactivity toward I/i carbohydrate antigens (31, 36, 37). There was a slightly greater proportion of intact (unmutated) AVY motifs in RA donors compared to healthy controls, in sequences of either the IgG or IgM isotype; although this effect was weak and only passed the threshold of significance for sequences of the IgM isotype (Figure 2D). The Asn-X-Ser N-glycosylation site (NHS) in the CDR2 region is associated with binding to commensal bacteria by innate like B cells (38) and is usually mutated in IgG^+ve^ B cells (35). In RA patients the proportion of IGHV4-34 IgG sequences where the NHS N-glycosylation motif was still intact was significantly higher in both IgG and IgM isotype sequences compared to healthy control donors (Figure 2E).

### IgG^hypoM^ Sequences Are Polyclonal

**Figure 3:**
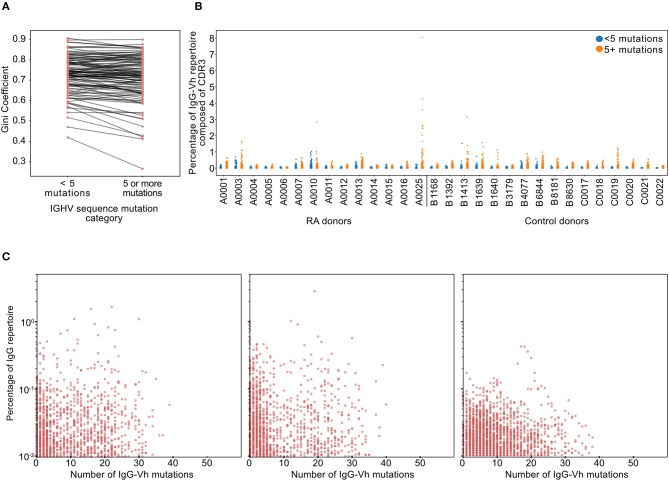
**(A)** Gini coefficients of IgG sequences for each RA donor from cohort 2 (*n* = 113). Gini coefficients are a measure of inequality of distribution, where a value of 0 indicates perfect equality (all IgG clonotypes of equal proportion). The Gini coefficient was calculated independently for hypomutated (fewer than 5 mismatches) or hypermutated (5 or more mismatches) sequences to compare the degree of clonal expansion in each category. **(B)** Percentage of the IgG-Vh repertoire composed of unique clonotypes from ERA patients and healthy controls (cohort 1, *n* = 14 + 16, respectively), with sequences split into hypermutated (5 mismatches or more) and hypomutated (fewer than 5 mismatches). For this analysis, clones were defined as having identical CDR3 sequences, sharing predicted V segment identity and having the same number of V segment mutations. **(C)** Scatterplots showing the percentage of the repertoire against the hypermutation rate for each clone for the three individual RA donors with the greatest frequencies of hypomutated B-cells (in cohort 1). Clone definition is the same as that used for **(B)**.

To confirm the polyclonal nature of the IgG^hypoM^ BCR sequences, an equality metric called the Gini coefficient was used to compare the degree of clonal expansion in the hypo- and hyper-mutated compartments of each patient's repertoire. The Gini coefficients of the IgG^hypoM^ and the IgG sequences with 5 or more mutations (defined here as hypermutated) from each patient within cohort 2 were similar, indicating that both components of the repertoire have similar clonotypic frequency structures (Figure 3A). We further investigated the degree of clonal dominance of IgG^hypoM^ from RA donors at the time of diagnosis and healthy controls derived from cohort 1 where an average of 200,000 BCRs were sequenced per donor. Concurring with two recently published reports on NGS of RA BCRs, the majority of RA patients and healthy controls expressed some clonotype frequencies >0.5% of the total sequence reads (39, 40) (Figure 3B). Within these repertoires, both hypermutated and IgG^hypoM^ sequences exhibited a similar distribution of clonotype frequencies, including a very large number of clonotypes with single reads (Figure 3C). To investigate the effect of clonal populations on the increased frequency of hypomutated IgG sequences in RA patients, we collapsed groups of reads sharing identical CDR3 amino acid sequences and with shared predicted V allele identity into clonal groups. With each clone only contributing once to the analysis, we again observed similar results indicating that the increased frequency of IgG^hypoM^ sequences could not be explained simply by the presence of plasmablasts or plasma cells (Supplementary Figure 5). One explanation for the higher frequency of IgG^hypoM^ in RA donors could be the failure of enzymes involved in somatic hypermutation (SHM), such as activation-induced cytidine deaminase (AID) or the subsequent mismatch repair enzymes. However, mutations were preferentially targeted to the same regions of the IgGHV segment in RA and control donors, and there were no inter-group differences in the targeting of mutation (10) prevalence of IgG^hypoM^ in RA donors did not result from AID or mismatch repair enzyme impairment or to the mono- or oligo-clonal expansions of IgG^+ve^ BCRs with few mutations Supplementary Figures 6, 7.

### The BCR^hypoM^ Are Expressed by IgG^+ve^CD27^–ve^ B Cells

**Figure 4:**
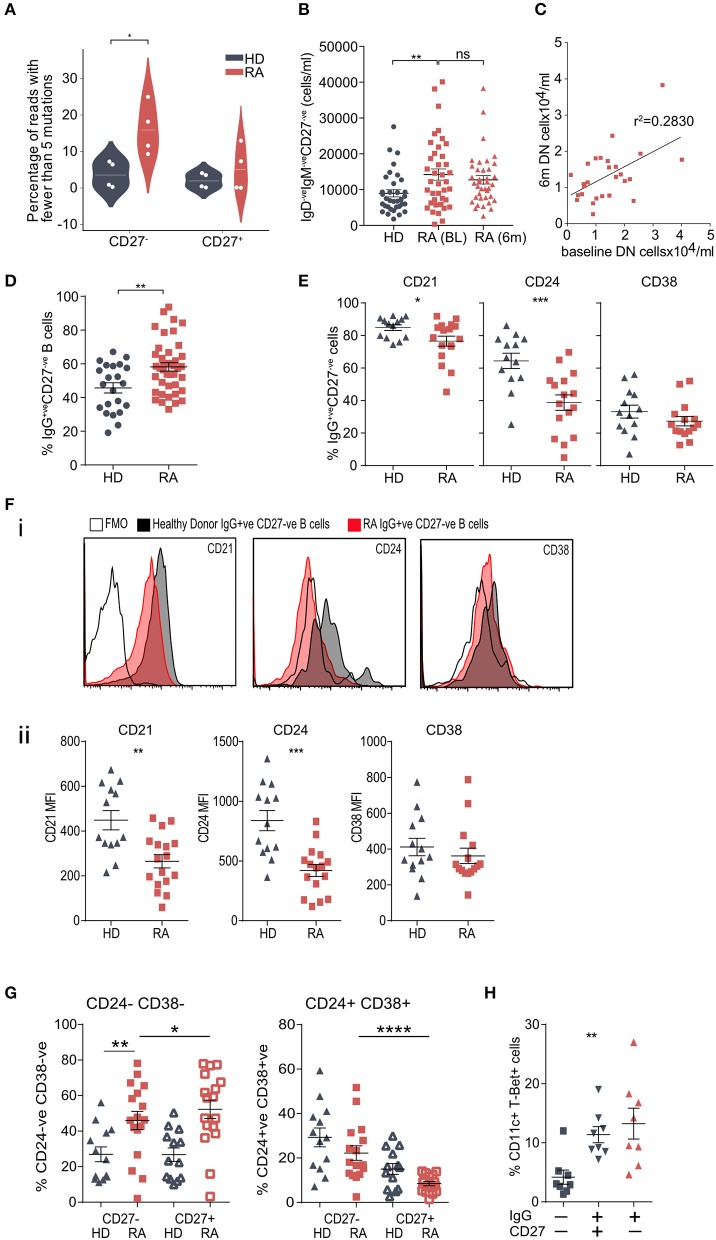
**(A)** Prevalence of hypomutated V segment sequences in CD27^+ve^ and CD27^−ve^ IgG^+ve^ B-cells from RA and control donors. White spots indicate individual data points for each donor (*n* = 4 donors per group). *P*-value calculated using Mann-Whitney test. **(B)** Whole blood was stained for flow cytometry to determine cell number. IgD^−ve^IgM^−ve^CD27^−ve^ B cells were gated and cells/ml calculated. *n* = 35 HD donors, *n* = 39 RA (0 m) donors, *n* = 38 RA (6 m) donors. *P*-value calculated using Mann-Whitney test. **(C)** Scatterplot of the frequencies of DN (IgD^−ve^IgM^−ve^CD27^−ve^) B cells from paired baseline and 6-month data from **(B)**, to determine if the frequency of DN B cells was correlated in the same patient 6 months following DMARD therapy. *n* = 27 patients. R^2^ value calculated using Pearson's correlation. **(D)** Cell frequencies of CD20^+ve^CD19^+ve^IgG^+ve^CD27^−ve^ B cells from PBMCs from control donors and RA patients assessed by flow cytometry. Cells were gated for CD20^+ve^CD19^+ve^IgG^+ve^ B cells then the proportion of CD27^−ve^ cells determined. *n* = 22 HD donors and 42 RA donors. *P-*value calculated using unpaired *T*-test. **(E)** Cell surface markers of IgG^+ve^CD27^−ve^ B cell population were analyzed by flow cytometry. Gating strategy is detailed in Supplementary Figure 8. *n* = 13 HD donors and 16 RA donors. *P*-values calculated using Mann-Whitney test. Further surface markers are shown in Supplementary Figure 8. **(F)** (i) Representative histogram plots of relevant fluorescence minus one (FMO) shown with open black line, healthy donor IgG^+ve^CD27^−ve^ (shaded black line) and RA IgG^+ve^CD27^−ve^ (shaded red line). (ii) Mean Fluorescence Intensity (MFI) data for individual samples was plotted for HD (gray) and RA (red) IgG^+ve^ CD27^−ve^ B cells. *P*-values were calculated using the Mann-Whitney test. *n* = 13 HD donors and 16 RA donors. **(G)** IgG^+ve^CD27^−ve^ B cells were also analyzed for dual staining of CD24 and CD38 by flow cytometry. Percentage was plotted for HD (gray) and RA (red) for both IgG^+ve^CD27^−ve^ (filled symbol) and IgG^+ve^CD27^+ve^ (open symbol) populations. For comparison of HD and RA IgG^+ve^ CD27^−ve^ populations *P*-values were obtained using Mann-Whitney test. For comparison of RA IgG^+ve^CD27^−ve^ to RA IgG^+ve^ CD27^+ve^ populations, *P-*values were obtained using Wilcoxon paired test. **(H)** Percentage of CD20^+ve^CD19^+ve^ CD11c^+^ T-Bet^+ve^ B cells within RA PBMC IgG^−ve^CD27^−ve^, IgG^+ve^CD27^+ve^ and IgG^+ve^CD27^−ve^ B cells. IgG^+ve^ B cell populations had a higher percentage of CD11c^+^ T-Bet^+ve^ B cells than IgG^−ve^CD27^−ve^, but there was no significant difference in the percentages of CD11c^+^ T-Bet^+ve^ B cells within IgG^+ve^ B cells. *n* = 8 RA donors. *P*-value calculated using Wilcoxon matched-pair test. *p < 0.05, **p < 0.01, ***p < 0.001, ****p < 0.0001.

The proportion of IgG^hypoM^ was significantly higher in the IgG^+ve^CD27^−ve^ B-cell population from RA patients than from either the IgG^+ve^CD27^+ve^ RA population or from the same population in the healthy controls (Figure 4A, Mann-Whitney U, *p* < 0.015). The absolute number of circulating peripheral blood double negative (IgM^−ve^IgD^−ve^CD27^−ve^) B cells in RA patients was also significantly increased at the time of diagnosis and did not change following 6 months of synthetic DMARD therapy (Figure 4B). Within individual RA patients, the number of double negative B cells at baseline and following 6 months of therapy was still clearly correlated, suggesting that they did not decrease significantly with DMARD treatment (Figure 4C; Supplementary Table 3C for patient data). Importantly the increase in double negative B cells was also reflected in a significant increase in the frequency of IgG^+ve^CD27^−ve^ B cells (Figure 4D). Circulating IgG^+ve^CD27^−ve^ B-cells from RA patients expressed less CD24, CD21, and CD38, but similar levels of CD73 and CD1c when compared to the same subset in healthy controls (Figures 4E,F; Supplementary Figure 8). B cells that lack expression of IgD, CD27, CD21, CD24, and CD38 are known as double negative 2 (DN2) B cells, whilst DN1 B cells express CD24 and CD38 (9). We observed a significant increase in the percentage of IgG^+ve^CD27^−ve^ B cells that were also negative for CD38 and CD24 staining, demonstrating that DN2 cells were increased in RA peripheral blood (Figure 4G). DN2 cells also express CD11c and T-bet and just over 10% of the IgG^+ve^CD27^−ve^ (and IgG^+ve^CD27^+ve^) B cells expressed both these markers (Figure 4H).

### IgG^+ve^CD27^–ve^ B Cells Are Enriched in the Synovium and Secrete TNF-alpha

**Figure 5:**
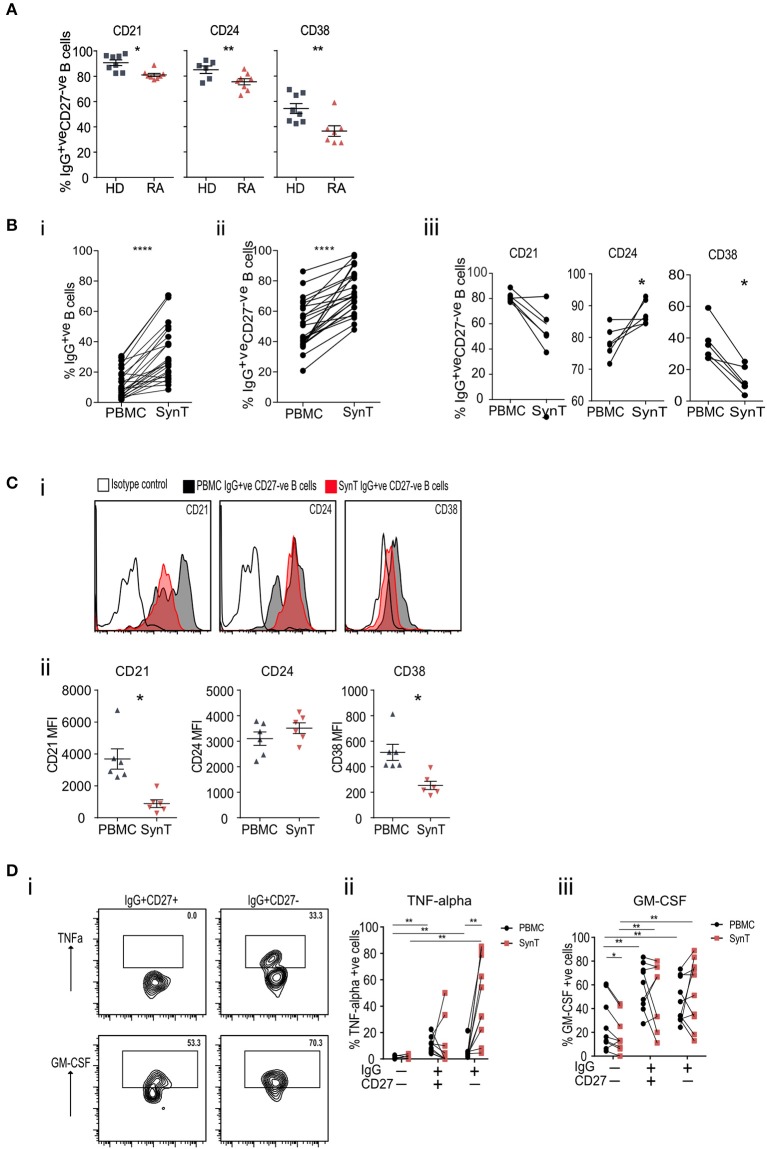
**(A)** Flow cytometry plots of peripheral blood IgG^+ve^CD27^−ve^ B cells stained for CD21, CD24, and CD38, taken from RA patients at the time of undergoing arthroplasty and compared to healthy donor PBMC. **(B)** (i) Flow cytometry of paired peripheral blood (PBMC) and synovial tissue (SynT) B cells from RA patients taken at the time of undergoing arthroplasty. The graph shows the percentage of IgG^+ve^ B cells within the CD20^+ve^CD19^+ve^ B cell population. (ii) Percentage of CD20^+ve^CD19^+ve^IgG^+ve^CD27^−ve^ B cells within the IgG^+ve^CD20^+ve^CD19^+ve^ B cell population. For (i) and (ii) *n* = 24 RA donors. *P*-value calculated using paired *t*-test. (iii) Paired RA PBMC and synovial tissue B cells within the IgG^+ve^CD27^−ve^ B cells stained for CD21, CD24 and CD38. *n* = 6 donors. *P*-value calculated using Wilcoxon matched-pair *T*-test. **(C)** Representative histogram plots of isotype control shown with open black line, PBMC IgG^+ve^CD27^−ve^ (shaded black line) and synovial Tissue IgG^+ve^CD27^−ve^ (shaded red line) (i). (ii) Mean Fluorescence Intensity (MFI) data for individual samples was plotted (ii) for PBMC (gray) and SynT (red) for IgG^+ve^ CD27^−ve^ B cells. *n* = 6 donors. *P*-values were calculated using the Wilcoxon paired test. Further surface marker data is shown in Supplementary Figure 9. **(D)** PBMC and synovial tissue cells were stimulated for 4.5 h with PMA, Ionomycin and Brefeldin A, then stained for intracellular cytokines. Representative flow cytometry plots showing the intracellular cytokine staining of synovial tissue IgG^+ve^CD27^−ve^ and IgG^+ve^CD27^+ve^ B cell subsets for TNF-alpha and GM-CSF (i). Pooled data for PBMC and Synovial tissue (ii) TNF-alpha and (iii) GM-CSF. Each point represents an individual patient sample. *n* = 9 donors. *P*-values calculated using Wilcoxon matched-pair test between tissue type or cell type. *p < 0.05, **p < 0.01, ***p < 0.001, ****p < 0.0001.

Utilizing blood samples from patients with established seropositive RA (ESRA) undergoing joint arthroplasty and comparing them to healthy donors (HD) further confirmed that IgG^+ve^CD27^−ve^ B cells expressed significantly less CD21, CD24, and CD38 (Figure 5A; cohort 4 in Supplementary Table 4). Compared to paired peripheral blood (PBMC) samples, the RA synovium was enriched for both IgG^+ve^ B cells (Figure 5Bi) and IgG^+ve^CD27^−ve^ B cells (Figure 5Bii). Specifically comparing paired PBMC and synovial IgG^+ve^CD27^−ve^ B cells showed a further reduction in the percentage of synovial IgG^+ve^CD27^−ve^ B cells that expressed CD21, CD38, and CD5 but a rise in CD24 expression (Figures 5Biii,C; Supplementary Figures 9B–D). When PBMC and synovial B cells were stimulated [with PMA/Ionomycin], synovial IgG^+ve^CD27^−ve^ B-cells expressed significantly more TNF-alpha than either the peripheral blood naïve (IgG^−ve^CD27^−ve^), memory (IgG^+ve^CD27^−ve^) or double negative B-cell populations and synovial naïve and memory B-cells. In contrast the production of GM-CSF was similar (Figure 5D). This data shows that IgG^+ve^CD27^−ve^ B-cells express significantly more IgG^hypoM^ and within the synovium they are primed to secrete more TNF than either memory or naive B cells.

### Peripheral Blood and Synovial B Cell Repertoires Are Distinct in RA

**Figure 6:**
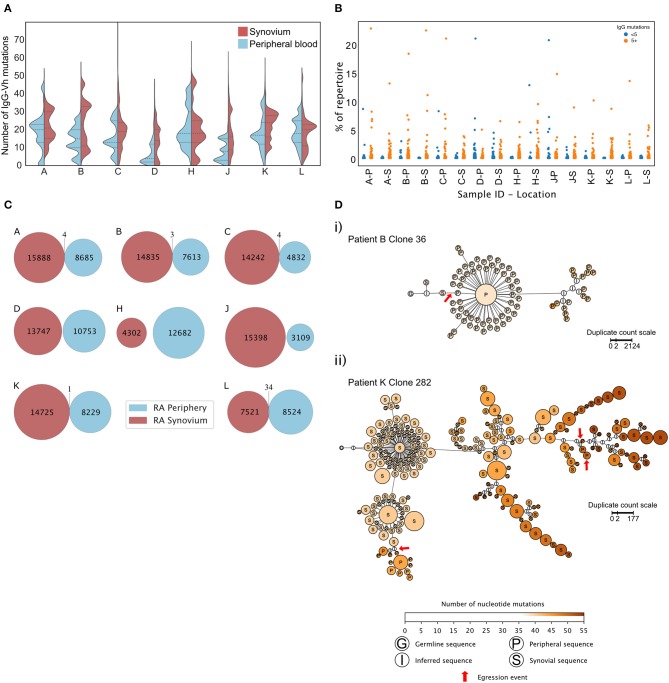
**(A)** Distributions of mutation counts from paired peripheral blood (blue) and synovial (red) IgG sequence repertoires from each of 8 RA patients with ESRA undergoing arthroplasty. The central horizontal line indicates the median of each distribution, with the upper and lower dashed lines representing the upper and lower quartiles, respectively. **(B)** Percentage of the repertoire composed of each CDR3 clonotype for paired synovial (S) and peripheral blood (P) samples from each of the 8 RA patients shown in **(A)** (cohort 4). **(C)** Repertoire overlap of synovial (red) and peripheral blood (blue) IgG repertoires of the RA patients. Each Venn diagram represents a single patient. The number of unique, non-singleton IgG sequences in the repertoire from each compartment is depicted at the center of each circle, and shared sequences are enumerated at the intersection between the two circles. Two shared sequences are considered identical if they possessed the same CDR3 nucleotide sequence and used the same V and J gene segments. **(D)** Lineage trees of B cell clones that show evidence of egression from the synovium into the periphery of RA patients, inferred for (i) clone 36 from patient B and (ii) clone 282 from patient K. Each node represents a unique non-singleton IgG sequence with the size of the node scales non-linearly in proportion to the number of sequence duplicated observed. The label at the center of each node represents the tissue origin of the sequence, and node color indicates the number of somatic mutations present in the clone sequence. Red arrows mark egression events from the synovium to the periphery. Lineage trees were inferred using PHYLIP v3.6 and plotted in Gephi v0.92.

As well as secreting cytokines, synovial B cells are reported to secrete autoantibodies that have undergone SHM within GC like structures (39, 41–43). Paired peripheral and synovial IgGHV sequences from RA patients undergoing arthroplasty were sequenced (Figure 6A). To avoid any potential confounding that may occur with increased levels of mRNA in plasma cells, only purified CD20^+ve^ B cells were analyzed. In six out of eight patients, the lower quartile of the distribution of somatic mutations was lower in the periphery than in the synovium. The mean number of mutations displayed by the peripheral B cell repertoire of patients (mean 15.27 ± 5.09) was lower than the mean number of mutations displayed by the paired synovial repertoires (mean 19.65 ± 2.96, paired *t*-test, *p* = 0.02). To exclude the effects of read duplication and to account for the presence of similar clones, the distribution of the number of IgGHV mutations was also plotted following single-linkage clonal clustering (Supplementary Figure 10). This removed the peaks associated high abundance sequence reads but in 7 of 8 patients, the distributions of the mean number of mutations per clonal cluster had a lower, more skewed distribution in peripheral blood than in the synovium. The RA synovium has been reported to harbor clonal expansions of B cells (39, 43). The percentage of the repertoire composed of each unique CDR3 clonotype was plotted, dividing the BCR sequences into those with greater or fewer than five mutations relative to the predicted germ line sequence (Figure 6B). No clear preference was noted within either the hypermutated or IgG^hypoM^ sequences in terms of CDR3 clonal frequencies with all four populations expressing CDR3 clonal frequencies >0.5% of the IgG^+ve^ B cell repertoire. A caveat to these observations was that, due to the smaller number of B cells purified from the synovium of ESRA patients, we utilized the Smart-seq2 protocol to amplify BCR RNA from both the blood and synovial samples (15). This afforded a greater opportunity for amplification bias, which may have increased the relative frequencies of abundant clones.

Finally, it was important to determine if RA patients shared BCR sequences that may point to particular pathogenic clonal expansions. The term “public sequence” is used to describe similar or identical T cell receptor (TCR) or BCR sequences which may arise in different individuals, indicative of a convergent immune response in different individuals to a common antigenic stimulus (44). This may have particular relevance for autoimmune mediated tissue damage including RA. We hypothesized that identical CDR3 sequences would be found in more than one patient in newly diagnosed DMARD naïve RA patients (cohort 1). Instead, we found that the degree of sharing of clonal sequence clusters between the peripheral blood repertoires of RA donors was no greater than between healthy donors (Supplementary Figure 11).

Finally, to ask if the IgG^hypoM^ sequences arose in the synovium, the frequency of identical CDR3 sequence clones present in the blood and synovium of the same RA patient was assessed. Very few CDR3 sequences were present in both the blood and synovium of the same patient (Figure 6C). Where overlap was seen (in the repertoires of 5 RA patients), it made up <0.1% of the synovial repertoire. Clonal lineage analysis of all shared sequences was employed to detect egression events from the synovial compartment. Only two lineage trees, from patient B and patient K, showed evidence of egression events from the synovium to the periphery (Figure 6D), with the other sharing events feasibly explained by index misassignment in sequencing. Given the extremely low support for egression events from the synovium, there is no evidence to support the hypothesis that the IgG^hypoM^ sequences in the peripheral blood B cell repertoires arise within the inflamed synovial joint.

## Discussion

This is the largest study to date, utilizing NGS, to examine the IgGHV repertoires of over 150 RA and 15 Sjögren's syndrome patients. RA patients express significantly more hypomutated BCRs within IgG^+ve^CD27^−ve^ B cells. In patients with established RA, IgG^+ve^CD27^−ve^B cells are enriched in the synovium, where they secrete TNF upon activation. The presence of IgG^hypoM^ sequences was also seen in patients with Sjögren's syndrome, indicating that this maybe a general feature of dysregulated B cell homeostasis in human autoimmunity. Significantly more IgGHV express a poorly mutated IGHV4-34 allele, which is known to be self-reactive in the germ line configuration. We hypothesize that auto-antibodies capable of driving chronic inflammation may arise from both peripheral and synovial B cells expressing IgG^hypoM^, via the formation of immune complexes and pro-inflammatory cytokines. We further hypothesize that following B cell depletion therapy, the frequency of IgG^hypoM^ would gradually increase to a critical threshold prior to a flare of synovitis. Future studies will confirm if the IgG^hypoM^ bind auto-antigens and their pattern of re-emergence following BCDT.

The stimuli driving the development of a high frequency of class switched IgG^hypoM^ in both RA and Sjögren's syndrome patients, is currently unknown. Central and peripheral tolerance checkpoints are known to be defective in RA patients, leading to the accumulation of naïve autoreactive B cells in the periphery (45). Activation of naïve B cells, facilitated by T cell help and/or TLR ligands, induces class switching out-with the germinal center (46). An extrafollicular response has been shown to drive the expansion of autoreactive barely mutated B cells in mice, that have the potential to cause arthritis or systemic lupus erythematosus (SLE) (47). In addition, humans with SLE have significant expansions of poorly mutated IgG VH4-34 at the time of disease flares (11). The high frequency of IgG^hypoM^ in RA patients suggests that they may have arisen from an ongoing extrafollicular response.

DN2 B cells may function as extrafollicular antibody secreting precursor cells, that arise from activated naïve B cells and have fewer somatic mutations within the BCR variable region than IgG^+ve^CD27^+ve^ memory B cells (9). DN2 cells also express CD11c and the transcription factor T-bet and are greatly expanded in patients with active SLE (10). In RA patients we observed a significant increase in peripheral blood IgG^+ve^CD27^−ve^CD24^−ve^CD38^−ve^ B cells. CD21 expression on IgG^+ve^CD27^−ve^ B cells was reduced compared to healthy controls. Whilst this indicates that DN2 cells are increased in RA patients we cannot say if IgG^hypoM^ are also definitively derived from DN2 B cells and future studies will address this.

A previous report alludes to the loss of clonally expanded populations of B cells from the peripheral blood into the synovium at the time of RA onset (40). Assessing a greater number of IgG sequences at diagnosis, we observed expanded populations of B cells in both RA and healthy controls, making it difficult to see how dominant BCR clones could predict the onset of RA in at-risk individuals. In addition, there was no greater degree of sharing of clonal sequence clusters between RA patients, when compared to healthy controls, which does not support the paradigm that RA is driven by a specific repertoire of pathogenic B cell clones. Repertoire overlap analysis also demonstrated a very low number of multi-compartmental IgG sequences when compared to the overall size of either repertoire, suggesting that the peripheral and synovial B cell repertoires are quite distinct within and between patients, with only low levels of sharing of identical clones between the compartments. The cause for this may, as previously reported, arise from the migration of peripheral blood B cells into the synovium (40). As synovial B cells express distinct chemokine receptors from peripheral B cells, they may be sequestered within the synovium and rarely be observed in the peripheral blood again (48). In line with this, we observed only two B cell clones that showed evidence of a migration event from the synovium into the peripheral blood, indicating that such events are rare and not the source of the peripheral blood IgG^hypoM^ B cells.

Over time an increasing number of RA patients are failing to respond to multiple synthetic and biologic DMARD therapies. Future studies will explore if these refractory RA patients express more IgG^hypoM^ double negative B cells and if their emergence following BCDT foretells clinical disease relapse.

## Data Availability Statement

The raw data supporting the conclusions of this article will be made available by the authors, without undue reservation, to any qualified researcher.

## Ethics Statement

The use of human samples for cohorts 1, 3, and 4 was approved by the South East Scotland Bioresource NHS Ethical Review Board (Ref. 15/ES/0094). Ethical permission to collect samples donated from the SERA inception (cohort 2) was approved by the West of Scotland Local Research Ethics Committee (Ref. 10/S0703/4). The patients/participants provided their written informed consent to participate in this study.

## Author Contributions

MG, GC, and DG designed the experiments, analyzed the data, and wrote the manuscript. MG and GC consented and collected samples. GC, KM, and CG carried out experiments. GC, LC, SG, and KM undertook the data analysis. HJ and SB contributed to clinical data and/or sample collection. IM and HJ reviewed the manuscript. IM managed the SERA inception cohort.

### Conflict of Interest

The authors declare that the research was conducted in the absence of any commercial or financial relationships that could be construed as a potential conflict of interest.
