# Supplementary material for: In Human Autoimmunity, a Substantial Component of the B Cell Repertoire Consists of Polyclonal, Barely Mutated IgG+ve B Cells
Source: Front Immunol. 2020 Mar 20;11:395. doi: 10.3389/fimmu.2020.00395 (PMC7099054; doi:10.3389/fimmu.2020.00395)
Supplement: Supplementary file 1 [file Table_1.pdf]

Supplementary Table 1

Cohort 1: Newly diagnosed RA and healthy controls.

	Cohort 1 ERA (n=14)	Cohort 1 HD (n=16)
Age (year, IQR)	52.6 (36;66.5)	53.1 (43.5;60.3)
Female (number)	11 (78.6%)	10 (62.5%)
CCP (IU/ml, IQR)	290.8 (94;503.3)	N/A
RF (IU/ml, IQR) *[1]	86.4 (15.9;67.9)	N/A
Baseline DAS28 (IQR) *[2]	5.4 (4.8;6.4)	N/A
Follow up DAS28 at 6 months (IQR) *[3]	4.1 (2.8;5.6)	N/A

*[1]n=9, *[2]n=10, *[3]n=9

Supplementary Table 2

Cohort 2:Scottish Early Arthritis (SERA) study

(i) Baseline data

	SERA study (n=113)
Age (year, IQR)	61.9 (53.6;71.1)
Female (number)	65 (57.5%)
CCP (IU/ml, IQR) *[1]	210.2 (5.7;340)
RF (IU/ml, IQR) *[2]	175.2 (15;348.5)
Baseline DAS28 (IQR)	5.0 (3.8;6.1)
Follow up DAS28 at 6 months (IQR)	3.8 (2.3;5.3)

*[1]n=97, *[2]n=64

(ii) SERA: 6 months post DMARD treatment

	SERA study (n=12)
Age (year, IQR)	64.6 (54.9;79.7)
Female (number)	8 (66.7%)
CCP (IU/ml, IQR)	186.9 (38;340)
RF (IU/ml, IQR)*[1]	231 (20;601)
Baseline DAS28 (IQR)	5.1 (3.6;6.4)
6 month DAS28 (IQR)	4.9 (3.3;6.7)
DMARD treatment	
- Methotrexate	10
- Hydroxychloroquine	4
- Sulfasalazine	5
- Leflunomide	1
Number of concurrent DMARDS	
- One	7
- Two	2
- Three	3

*[1]n=3

Supplementary Table 3A

Cohort 3:Patients with established RA

that had failed synthetic DMARD therapy

	Cohort 3<br>N=16
Age (years, IQR)	52.6 (37;67.8)
Gender (female)	12 (75%)
CCP (IU/ml, IQR)*1	232.1<br>(61.8;405)
RF (IU/ml, IQR) *2	69.3 (10.6;131)
DMARD (current/previously failed)	
- Methotrexate	16
- Hydroxychloroquine	12
- Sulfasalazine	15
- Leflunomide	10
- Gold	2
- Cyclosporin	1
Number of previous Biologic DMARDs	
- None	1
- One	6
- Two	6
- Three	2
- Four	1
Biologic agents (current/previously failed)	
- Certolizumab	14
- Etancercept	6
- Adalimumab	1
- Rituximab	4
- Tocilizumab	2
- Abatacept	1

*1N=14, *2N=11

Supplementary Table 3B

Sjogren's Syndrome patients	(n=14)
Age (year, IQR)	50.5 (38;64)
Female [number (n)]	12 (87%)
Positive for either anti Ro/La	100%
RF (IU/ml, IQR) n=12 patients	83 (2.7;290)
Anti-dsDNA (IU/ml) n=1 patient	38.7

Supplementary Table 3C

	RA cohort for fig 4B<br>Baseline (n=39)<br>6m (n=38)	RA cohort for fig 4C<br>Baseline and 6m<br>(n=27)
Age (year, IQR)	55.1 (28.8;67.3)	56.2 (43.5;68.2)
Female (number)	25 (64.1%)	17 (63%)
CCP (IU/ml, IQR)	154.1 (6.4;340) *1	112.7 (5.3;288) *4
RF (IU/ml, IQR)	214.3 (20;277) *2	229.3 (20;442) *5
Baseline DAS28 (IQR)	5.0 (3.8;5.9)	5.0 (4.1;5.9)
DAS28 at 6 months (IQR)	3.9 (2.7;4.9) *3	3.9 (2.3;5.3)*6

*1n=30, *2n=31, *3n=35, *4n=19, *5n=23, *6n=24,

Supplementary Table 4

Cohort 4: Matched synovial and peripheral blood samples from joint arthroplasty

	Median 65, IQR 57.25-67
Sex	6 Female, 2 Male
Serum CCP and RF	8/8 CCP positive<br>6/6 RA positive (data not available for 2 samples)
Smoking	4/5 smokers (data not available for 3 samples)
Synovial sampling site	7 knee, 1 hip
DMARD therapy at time of sampling	Sulphasalazine 1<br>Tocilizumab 1<br>NIL 1<br>Prednisolone 1<br>Sulphasalazine/Methotrexate 2<br>Prednisolone/Azathioprine 1<br>Methotrexate/Humira 1

Supplementary Table 5

Supplementary table 5: Methods summary for cell preparation and B cell repertoire amplification

Cohort	Description	Cell site and RNA preparation	Amplification method	Isotype(s) amplified*
1	Early RA (ERA) and control donors, n=14 and n=16	PBMC purification, CD19+MACS	PCR amplification using IGHV FR1 primers, IGHC primers	IgM & IgG
2	Early RA (ERA), n=113	Paxgene tubes – 3mL whole blood.	PCR amplification using IGHV FR1 primers, IGHC primers	IgG
3	Established RA (ESRA), n=16	PBMC purification, CD19+MACS	PCR amplification using IGHV FR1 primers, IGHC primers	IgG
4	Paired PBMC and synovial B cells and CD27 sorted B cells	CD20+, IgG+, CD27+or CD27-	SMARTseq2 whole-transcriptome amplification followed by PCR amplification using IGHV FR1 primers, IGHC primers	IgG

*for primer information, see supplementary figure 1

SupplementaryTable 6

Supplementary Table 6: Antibody reagents for flow cytometry	
Antibody Reagent	Clone Reference
CD19-PE/Cy5	HIB19
CD20-AF700	2H7
CD21-BV421	B-ly4
CD27-APC	M-T271
CD38-BV510	HIT2
CD73-BV605	AD2
CXCR3-PE	IC6
GMCSF-PE/CF594	BVD2-21C11
CD5-PE/Cy7	L17F12
CD11C-BV711	3.9
CD21-PE/Cy7	Bu32
CD21-PerCP/Cy5.5	Bu32
CD24-BV711	ML5
CD24-FITC	ML5
CD38-BV605	HIT2
CD40-APC/Cy7	5C3
CD86-BV711	IT2.2
CD95(FAS)-BV510	DX2
CD254(RANKL)-PE	MIH24
HLA-DR-APC/Cy7	L243
IgD-APC/Cy7	IA6-2
T-Bet-PE/Cy7	4B10
TNFα-BV510	Mab11
CD1c-PerCP/eFluor710	L161
CD20-eFluor450	2H7
CD307d(FcRL4)-PerCP/eFluor710	413D12
IgG-FITC	IS11-3B2.2.3
IgG-PE	IS11-3B2.2.3
