# Supplementary material for: In Human Autoimmunity, a Substantial Component of the B Cell Repertoire Consists of Polyclonal, Barely Mutated IgG+ve B Cells
Source: Front Immunol. 2020 Mar 20;11:395. doi: 10.3389/fimmu.2020.00395 (PMC7099054; doi:10.3389/fimmu.2020.00395)
Supplement: Supplementary file 2 [file Data_Sheet_1.pdf]

Scottish Early Rheumatoid Arthritis Inception cohort (SERA)

Steering Committee Members: Duncan Porter[1] and Iain McInnes[1] (Chief Investigators); David Reid[2], Stuart H. Ralston[3], Neil Basu[2], Leon Collis[4], Carl S. Goodyear[1], Janet Liversidge[2]

Study Team: Caron Paterson[1], Jane Hair[5], Sharon Kean[6], Ashley Gilmour[1]

1. University of Glasgow, Institute of Infection, Immunity and Inflammation, Glasgow UK
2. School of Medicine and Dentistry, University of Aberdeen, Aberdeen, UK
3. Rheumatology and Bone Diseases Unit, Centre for Genomic and Experimental Medicine, MRC Institute of Genetics and Molecular Medicine, Western General Hospital, University of Edinburgh, UK
4. Pfizer Inc. Cambridge, USA
5. NHS Greater Glasgow & Clyde Bio-repository, Pathology Department, Southern General Hospital, Glasgow, UK
6. University of Glasgow, Robertson Centre for Biostatistics, Institute of Health and Wellbeing, Glasgow UK

Investigators:

Margaret Duncan, Ayr Hospital

Susan Fraser, Southern General Hospital, Glasgow

Lisa Hutton, Inverclyde Royal Hospital

John Harvie, Raigmore Hospital, Inverness

Vinod Kumar, Ninewells Hospital, Dundee

Mike McMahon, Dumfries & Galloway Royal Infirmary

Robin Munro, Wishaw General Hospital

John Larkin, Victoria Infirmary Glasgow

Neil McKay, Western General Hospital, Edinburgh John

McLaren, Whyteman's Brae Hospital, Fife David M Reid,

Aberdeen Royal Infirmary

Duncan Porter, Gartnavel General Hospital, Glasgow

Ruth Richmond, Borders General Hospital, Melrose

Gillian Roberts, Vale of Leven Hospital

Sarah Saunders, Glasgow Royal Infirmary

Hilary Wilson, Stobhill Hospital, Glasgow
