# Supplementary material for: In Human Autoimmunity, a Substantial Component of the B Cell Repertoire Consists of Polyclonal, Barely Mutated IgG+ve B Cells
Source: Front Immunol. 2020 Mar 20;11:395. doi: 10.3389/fimmu.2020.00395 (PMC7099054; doi:10.3389/fimmu.2020.00395)
Supplement: Supplementary file 3 [file Image_1.pdf]

Supplementary Figure 1

A

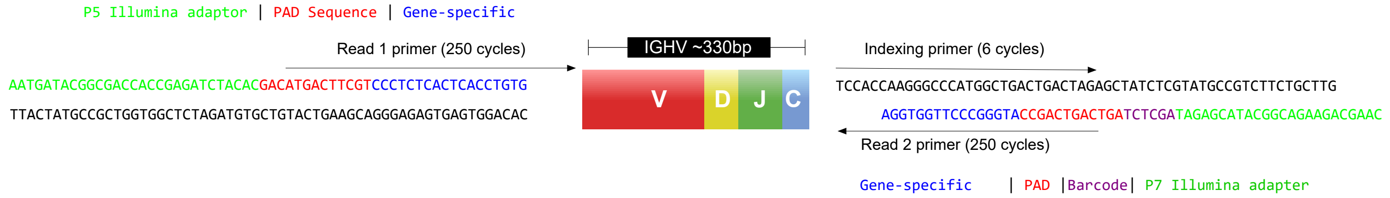

B

**IGHV FR1 forward amplification primers**	
hIGHV2.0	AATGATACGGCGACCACCGAGATCTACACGACATGACTTCGTCCCTCTCACTCACCTGTG
hIGHV2.1	AATGATACGGCGACCACCGAGATCTACACGACATGACTTCGTCCCTKAGACTCTCCTGTG
hIGHV2.2	AATGATACGGCGACCACCGAGATCTACACGACATGACTTCGTCCCTGARACTCTCCTGTG
hIGHV3.0	AATGATACGGCGACCACCGAGATCTACACGACACTACAGCGTAGACCCTCACRCTGACCT
hIGHV0.0	AATGATACGGCGACCACCGAGATCTACACGACATAGACCGGTTCAGTGAAGGTYTCCTGC
hIGHV1.0	AATGATACGGCGACCACCGAGATCTACACCGCCGGAAGCAGTGCTGAGGTGAAGAAGCCT
hIGHV1.1	AATGATACGGCGACCACCGAGATCTACACCGCCGGAAGCAGTYCAGGACTGGTGAAGCCT
hIGHV1.2	AATGATACGGCGACCACCGAGATCTACACCGCCGGAAGCAGTSCAGGACTGTTGAAGCCT
hIGHV4.0	AATGATACGGCGACCACCGAGATCTACACCATGTGCCTGTGTGTCCCTGAGACTCTCCTG
hIGHV4.1	AATGATACGGCGACCACCGAGATCTACACCATGTGCCTGTGTGTCTCTGARGATCTCCTG
hIGHV5.0	AATGATACGGCGACCACCGAGATCTACACCTTAGAGTCACGTCTCCTGCAAGGYTTCTGG
**IgGHC reverse primer**	
hIGGHCrev(INDEX1)	CAAGCAGAAGACGGCATACGAGATAGCTCTAGTCAGTCAGCCATGGGCCCTTGGTGG*A

Supplementary Figure 1

A: PCR amplicon sequence and sequencing strategy. Non-template encoded sequences are incorporated into amplicons during PCR as shown. Read 1 primer, Indexing primer and Read 2 primer locations are indicated by the black arrows.

B: Primer sequences for amplification of IgG and IgM repertoires. Primers contain a hexameric indexing sequence. For illustration only a single index is shown for each reverse primer.

Supplementary Figure 2

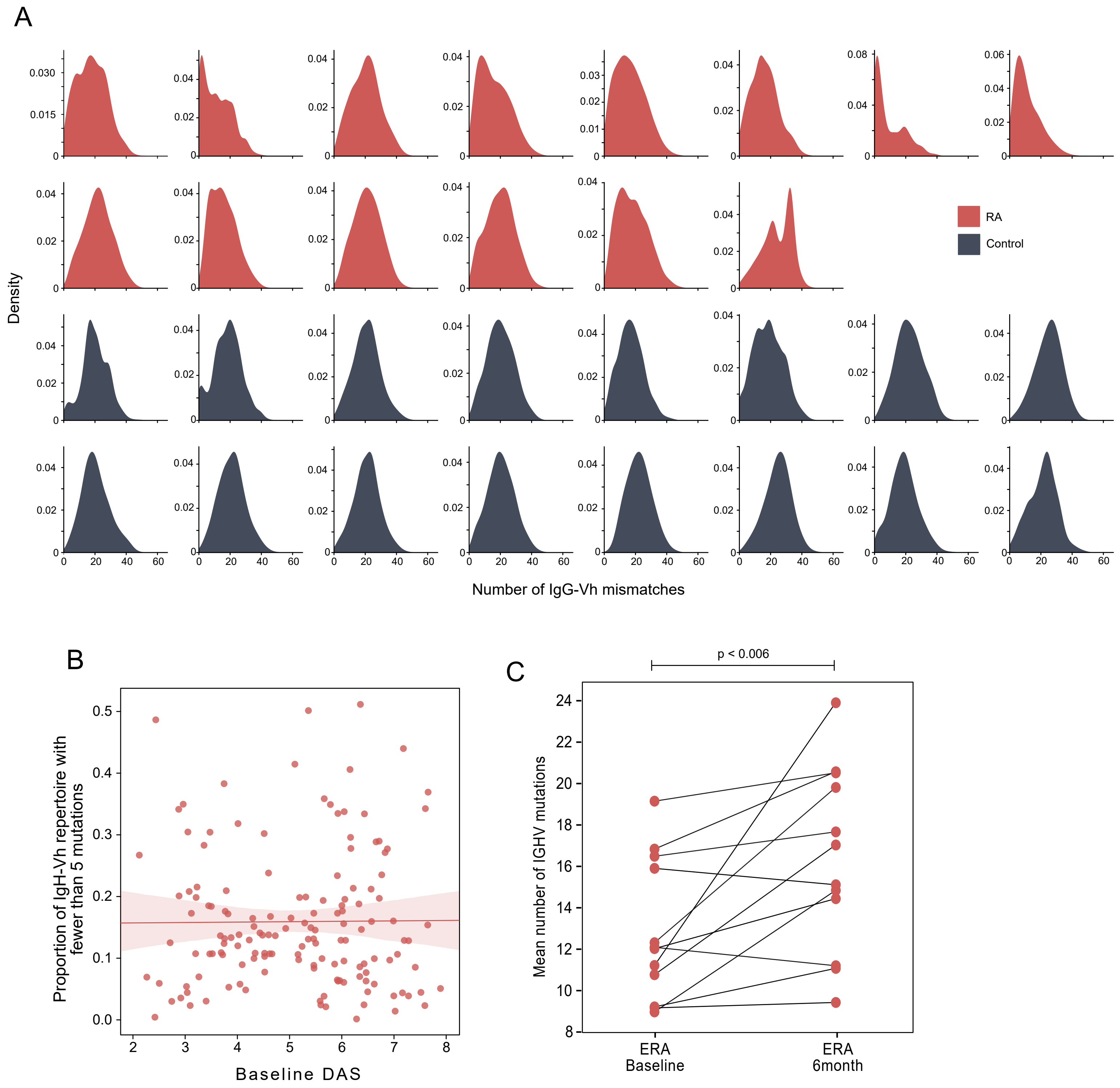

Supplementary Figure 2

A: Distribution of the number of IgG-Vh mismatches for each sequencing read of ERA (n=14) and control donors (n=16) from cohort 1. B: Linear regression of the proportion of the IgG-Vh reads from each repertoire against the disease activity score (DAS) at the time of diagnosis in cohort 2. Spearman correlation coefficient = -0.024832. The regression line is show as a solid red line, with the 95% confidence interval in indicated by the surrounding shaded area. C: Mean number of IgG-Vh mutations per read for paired ERA patients form cohort 2 at baseline and 6 months (n=12 donors). Lines connect the two measurement timepoints for each patient.

Supplementary Figure 2D

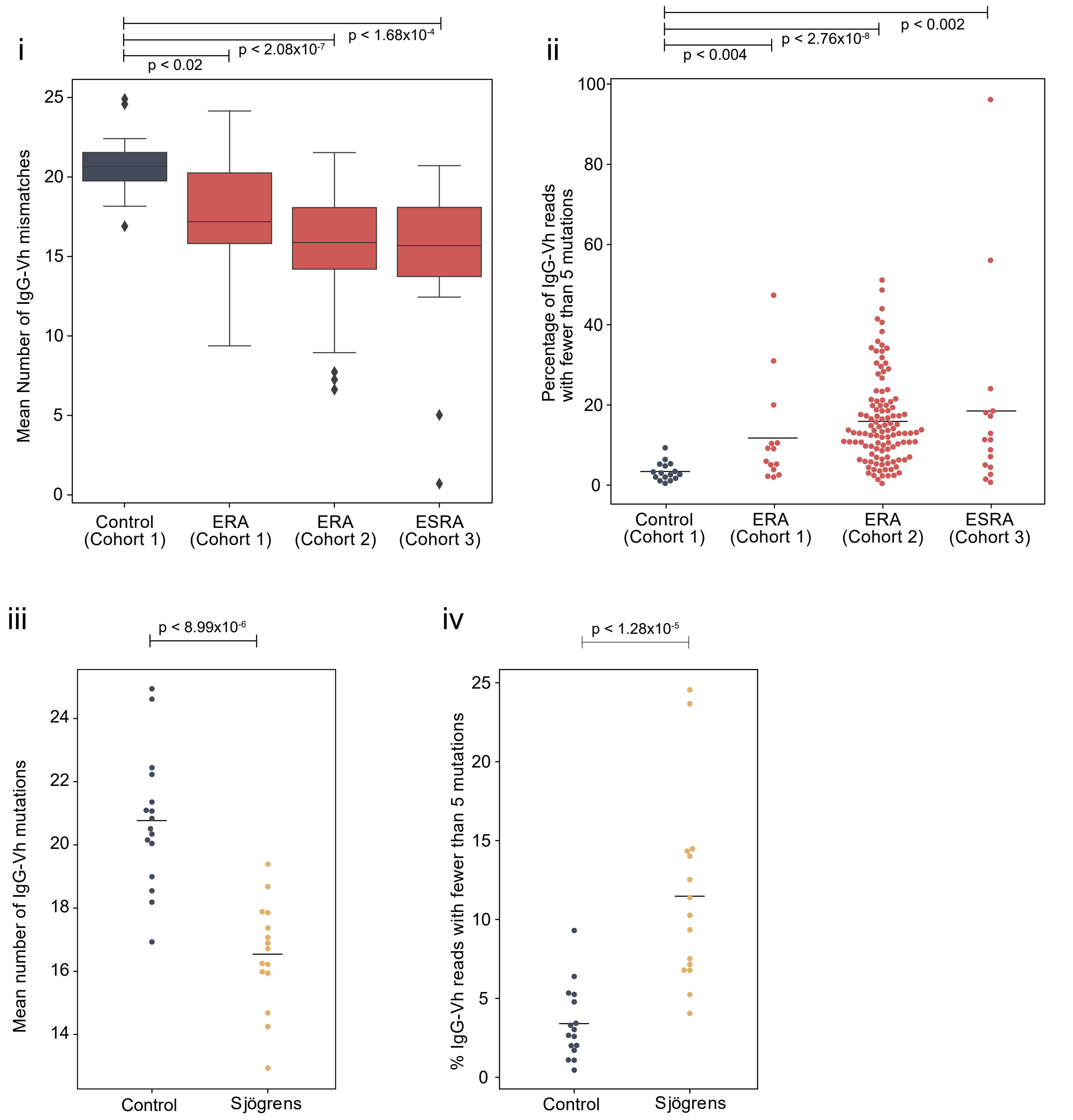

Supplementary Figure 2D: Frequency of IgG-Vh mutations in study cohorts

i. Mean IgG-Vh mismatches for control donors (n=16), ERA donors from cohorts 1 and 2 (n=14 and n=113 respectively) and ESRA from cohort 3 (n=16). P values are generated by Kruskal-Wallis test with Dunn's post-test to compare the median value for each RA group with the control donor group.
ii. Percentage of IgG reads with fewer than 5 mutations for healthy donors from cohort 1 (n=16), ERA donors from cohort 1 and cohort 2 (n=14 and n=113) and ESRA donors from cohort 3 (n=16).
iii. Mean number of mutations per IgG read for control donors from cohort 1 (n=16) and donors with Sjögrens syndrome from cohort 6 (n=15). Horizontal bars denote group arithmetic mean.
iv. Percentage of IgG reads with fewer than 5 mutations for control donors from cohort 1 (n=16) and donors with Sjögrens syndrome from cohort 6 (n=15). Horizontal bars denote group arithmetic mean.

Supplementary Figure 3

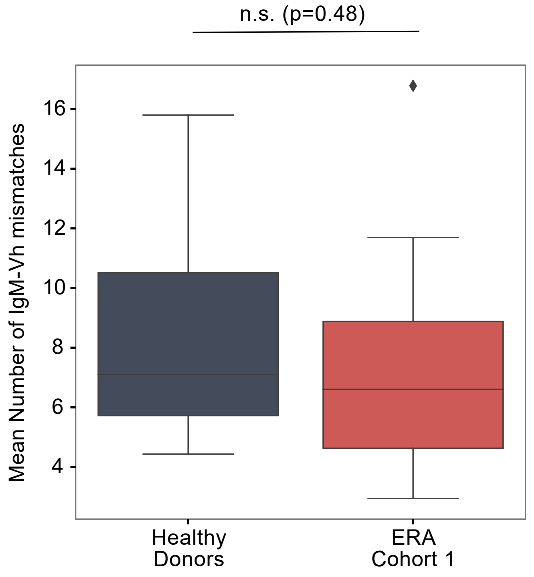

Supplementary Figure 3: Mean IgM-Vh mismatches for control donors (n=16) and ERA donors from cohorts 1 (n=14). The mean number of hypermutations for donors from each group were compared using Student's t-test (assuming unequal variances) (p=0.48).

Supplementary Figure 4

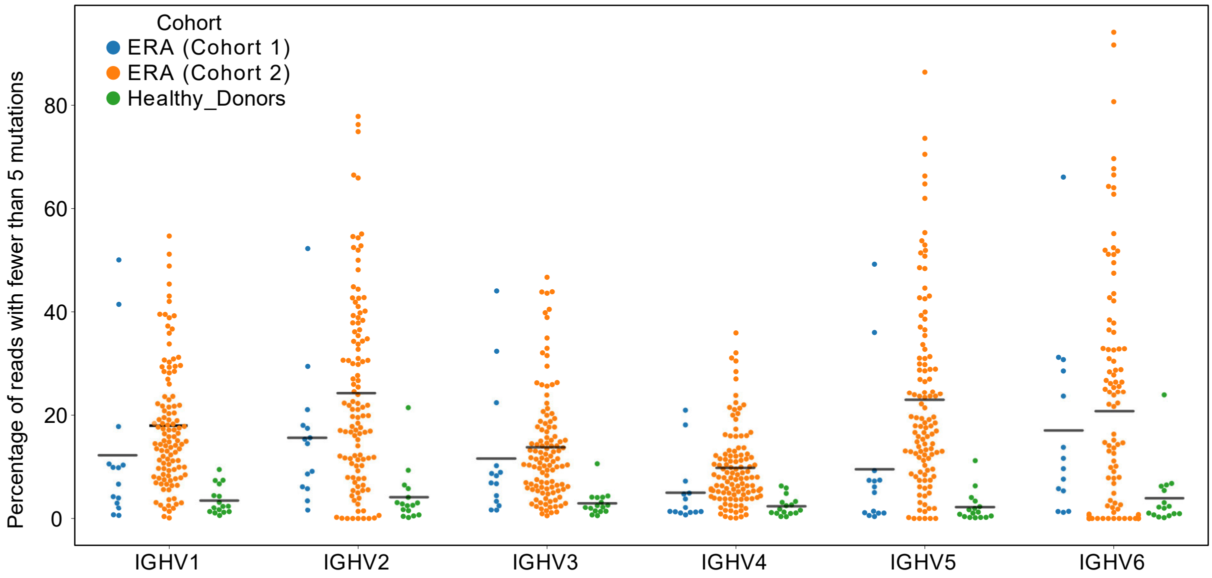

Supplementary Figure 4

Percentage of IgG reads from ERA and healthy donors that have fewer than 5 mutations, split by germline IGHV allele family. Only reads that map to the six most frequent IGHV families (IGHV1-6) are shown to ensure sufficient reads are available in each donor to be representative. Horizontal bars denote the group mean.

Supplementary Figure 5

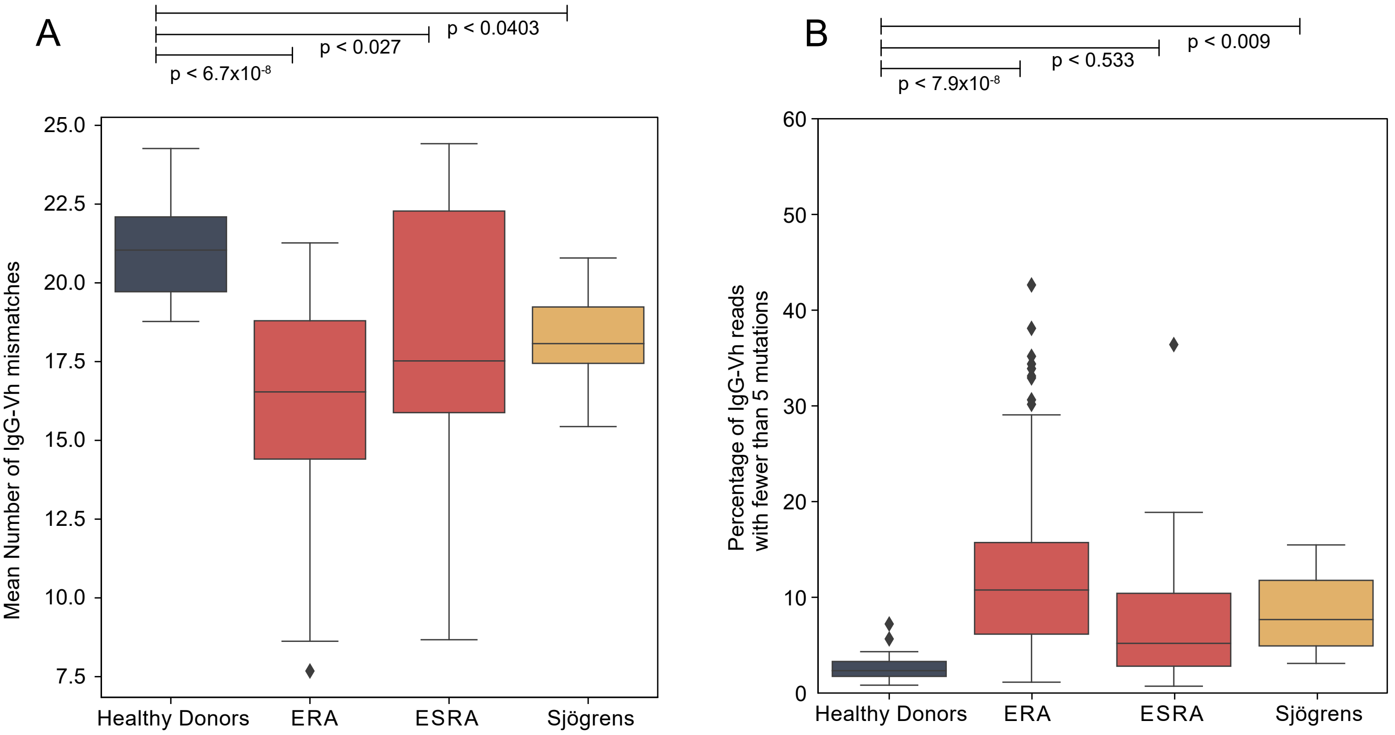

Supplementary Figure 5: Analysis of IgG-Vh mutation frequency from BCR sequence clones. Reads were dereplicated by grouping all reads sharing common CDR3 amino acid sequences and predicted germline V allele identity, with each individual clone retaining the mean mismatch frequency of all of the contributing reads.

A. Mean IgG-Vh mismatches for control donors (n=16), ERA donors from cohorts 1 and 2 combined (n=127) and ESRA from cohort 3 (n=16) and Sjogrens Syndrome donors (n=15).

B. Percentage of IgG reads with fewer than 5 mutations for control donors (n=16), ERA donors from cohorts 1 and 2 combined (n=127) and ESRA from cohort 3 (n=16) and Sjogrens Syndrome donors (n=15). P values are generated by Kruskal-Wallis test with Dunn's post-test to compare the median for each RA group with the control donor group.

P values are generated by Kruskal-Wallis test with Dunn's post-test to compare the median for each RA group with the control donor group.

Supplementary Fig 6

i) Early RA donors

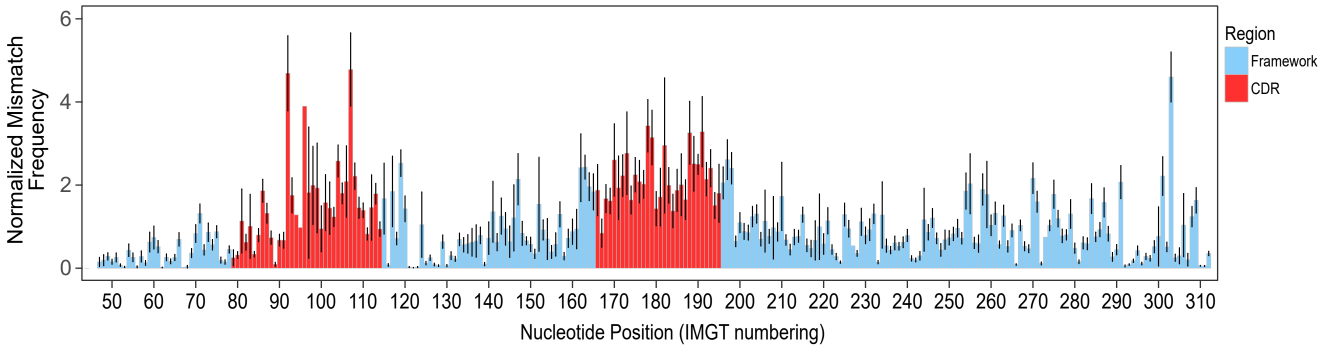

ii) Healthy control donors

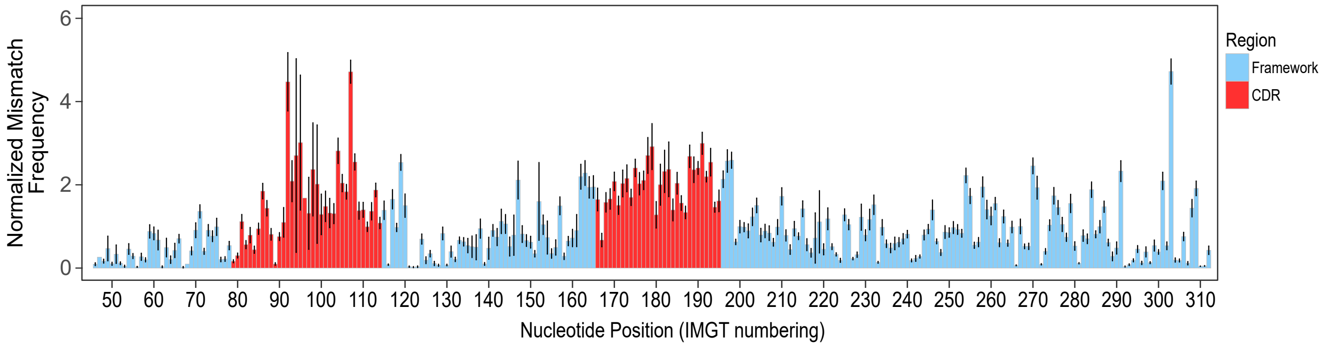

Supplementary Figure 6

Average frequency of mismatches along the V segment for IgG sequences i) RA and ii) healthy control donors form cohort 1 (n=14 & n=16 respectively). The normalised mismatch frequency is calculated as the number of times a mismatch appears at the IMGT position divided by the number of times the IMGT position appears within the aligned sequences of each patient. The group mean is calculated from individual patient frequencies at each IMGT position; error bars show 95% CI of the mean. Mean and 95% CI are normalized by the group mean for per-base mismatch frequency. Red bars show the CDR and blue bars show the FR regions.

Supplementary Figure 7

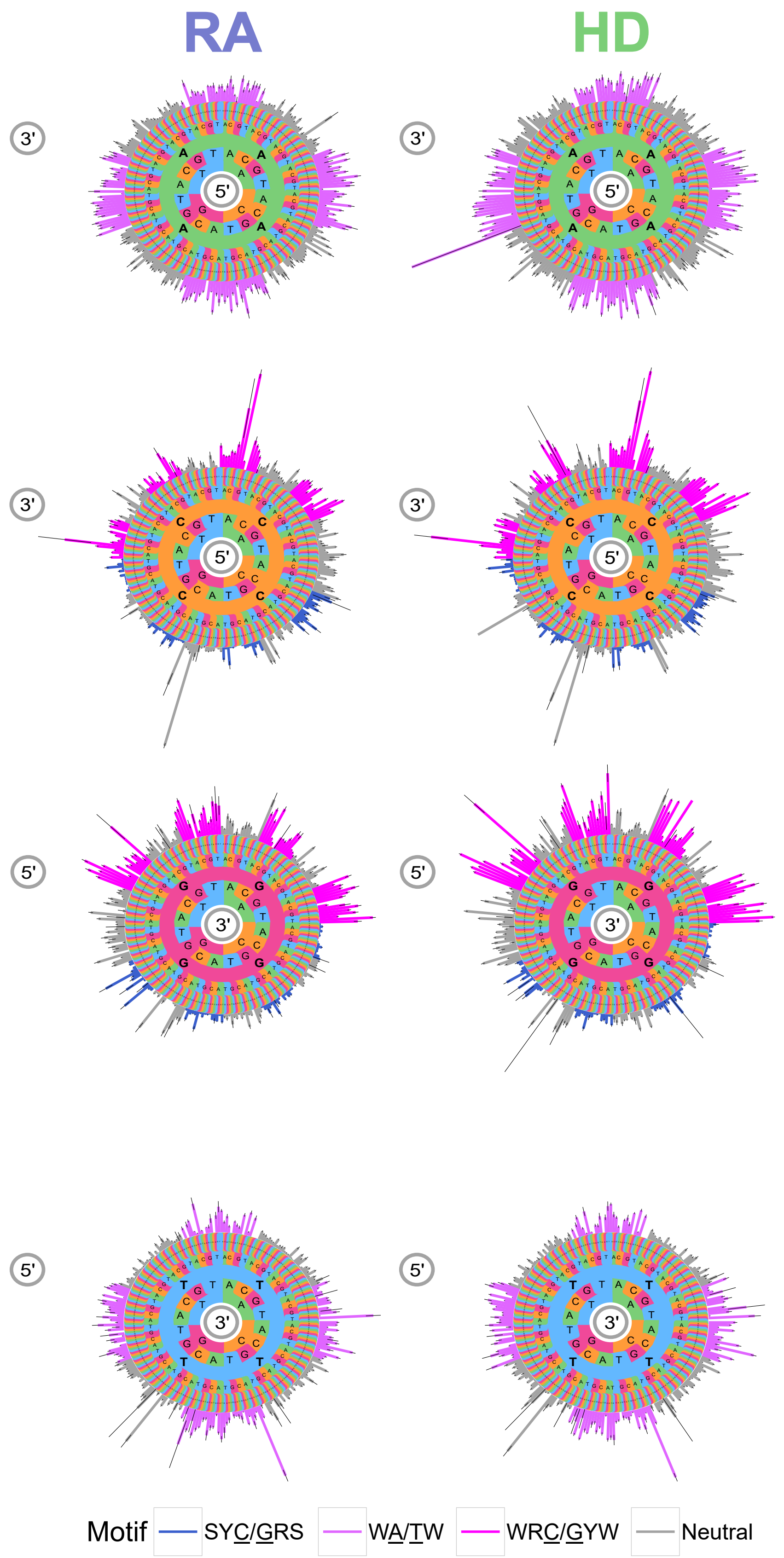

Supplementary Figure 7. Motif Mutabilities of All Mutations in IgG repertoires of individuals from cohort 1 (n=16 for HD and n=14 for RA). Analysis of motif mutability in all mutations to assess targeting by SHM and selection. Data are shown as hedgehog plots: Circular bar graphs show the group mutation frequency of the central nucleotide in a 5-nucleotide kmer. The central set of concentric circles shows the initial sequence of the 5-mer and the colour of the bars shows the motif identity (hotspots, coldspots, and neutral). Mutation frequency is calculated as the number of times the central nucleotide is mutated, divided by the number of times the kmer appears in a patient. Group frequencies are calculated as the mean of each patient kmer mutation frequency and normalized by the per-base mismatch frequency for that group. Error bars show the 95% CI of the mean. R = (A or G), S = (C or G), Y = (C or T), and W = (A or T)

Supplementary Figure 8

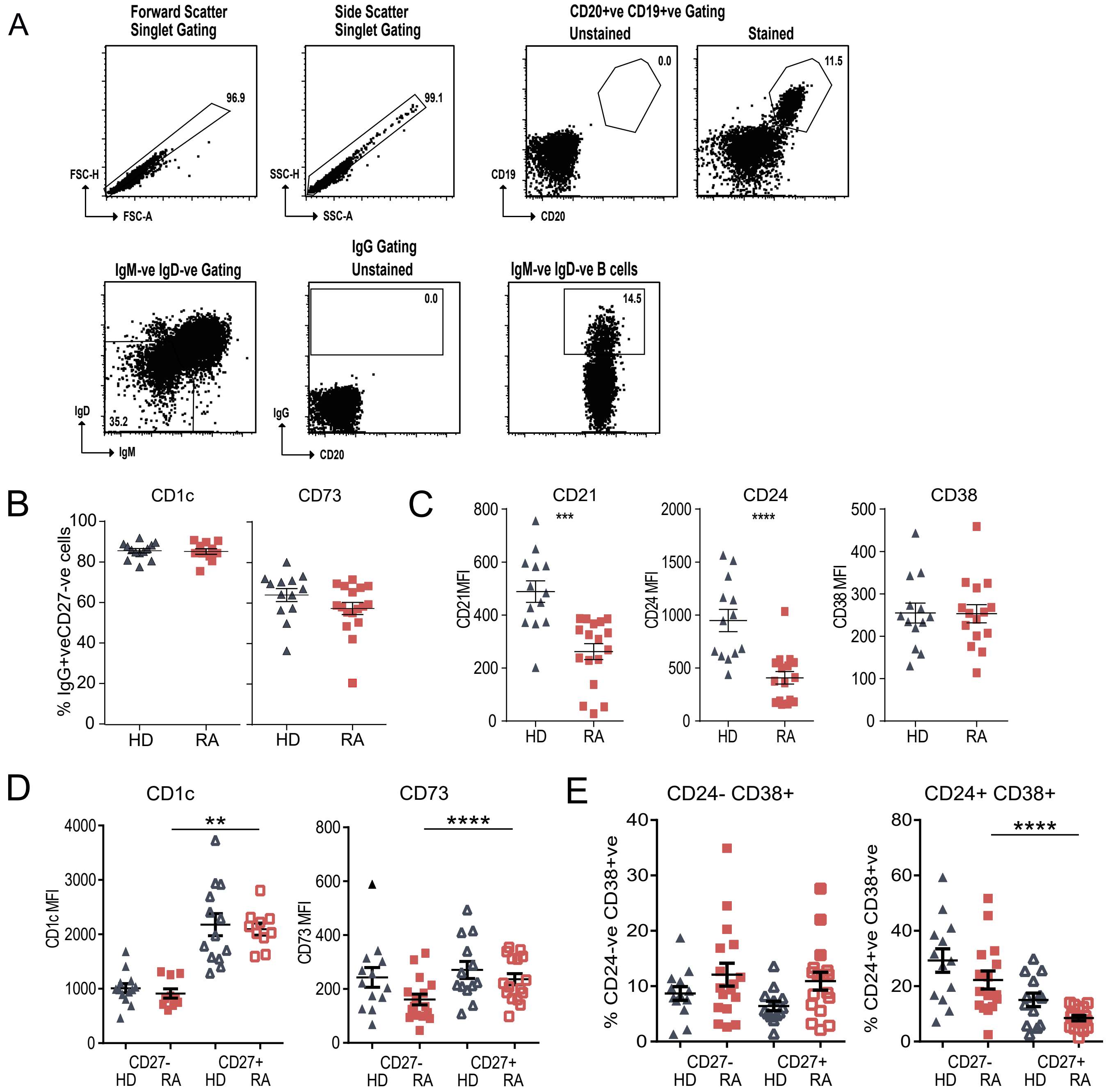

Supplementary Figure 8

A. Flow cytometry plots to show gating strategy for Figure 4. FSC and SSC singlet gates were applied before selecting CD19+ve CD20+ve cells. IgM-ve IgD-ve cells were then gated, followed by IgG positive gating.

B. Flow cytometry of IgG+ve CD27-ve cells showing cell surface markers in addition to those shown in Figure 4E (N=13 HD & 16 RA). HD are shown in grey and RA in red.

C. IgG+ve CD27+ve cells were also analysed for mean fluorescence intensity (MFI) of CD21, CD24 and CD38. HD are shown in grey and RA in red. P values were obtained using Mann-Whitney test.

D. Flow cytometry of additional surface marker MFI. HD (grey) and RA (red) for both IgG+veCD27-ve (filled symbol) and IgG+veCD27+ve (open symbol) B cells. For comparison of HD and RA IgG+veCD27-ve populations P values were calculated using the Mann-Whitney test. For comparison of RA IgG+ve CD27-ve to RA IgG+ve CD27+ve populations P values were obtained using Wilcoxon paired test.

E. Flow cytometry shown in Fig 4E-F was also analysed for dual staining of CD24 and CD38. Percentage was plotted for HD (black) and RA (red) for both IgG+ve CD27-ve (filled symbol) and IgG+ve CD27+ve (open symbol) populations. For comparison of HD and RA IgG+ve CD27-ve populations P values were obtained using Mann-Whitney test. For comparison of RA IgG+ve CD27-ve to RA IgG+ve CD27+ve populations, P values were obtained using Wilcoxon paired test.

Supplementary Figure 9

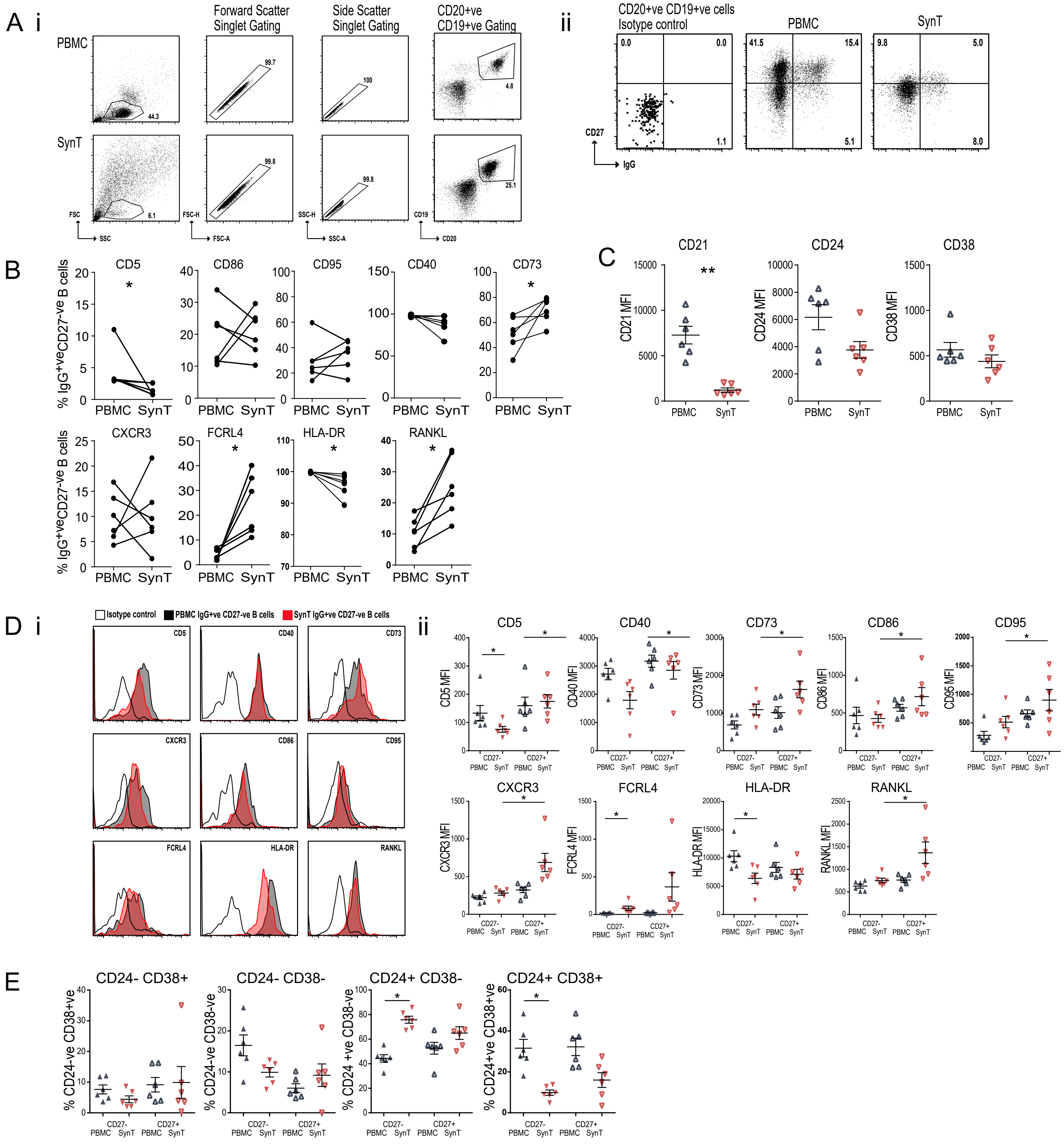

Supplementary Figure 9

A. Flow cytometry plots to show gating strategy for Figure 5. FSC and SSC singlet gates were applied before selecting CD19+ve CD20+ve cells (i). IgG+ve CD27-ve cells were then gated for as shown (ii).

B. IgG+ve CD27-ve cells were analysed using flow cytometry for additional surface markers. Percentage of each cell surface marker is shown for PBMC and Synovial Tissue. P value calculated using Wilcoxon paired T test

C. IgG+ve CD27+ve cells were also analysed for mean fluorescence intensity (MFI) of CD21, CD24 and CD38. PBMC are shown in grey and Synovial Tissue in red. P values were obtained using Wilcoxon test.

D. Cells stained in Fig 5B-C were stained for further cell markers. Representative histogram plots of isotype control (open black line), PBMC IgG+ve CD27-ve (shaded black line) and SynT IgG+ve CD27-ve (shaded red line) are shown in (i). Graphs in (ii) show mean fluorescence intensity (MFI) for each cell surface marker and plotted for PBMC (grey) and SynT (red) for both IgG+ve CD27-ve (filled symbol) and IgG+ve CD27+ve (open symbol) populations. P values were obtained using Wilcoxon matched-pairs test comparing IgG+ve CD27-ve PBMC and SynT or SynT IgG+ve CD27-ve and IgG+ve CD27+ve.

E. PBMC and Synovial Tissue were also analysed for dual staining of CD24 and CD38. Percentage was plotted for PBMC (grey) and SynT (red) for both IgG+ve CD27-ve (filled symbol) and IgG+ve CD27+ve (open symbol) populations. P values were obtained using Wilcoxon matched-pairs test comparing IgG+ve CD27-ve PBMC and SynT or SynT IgG+ve CD27-ve and IgG+ve CD27+ve.

Supplementary Figure 10

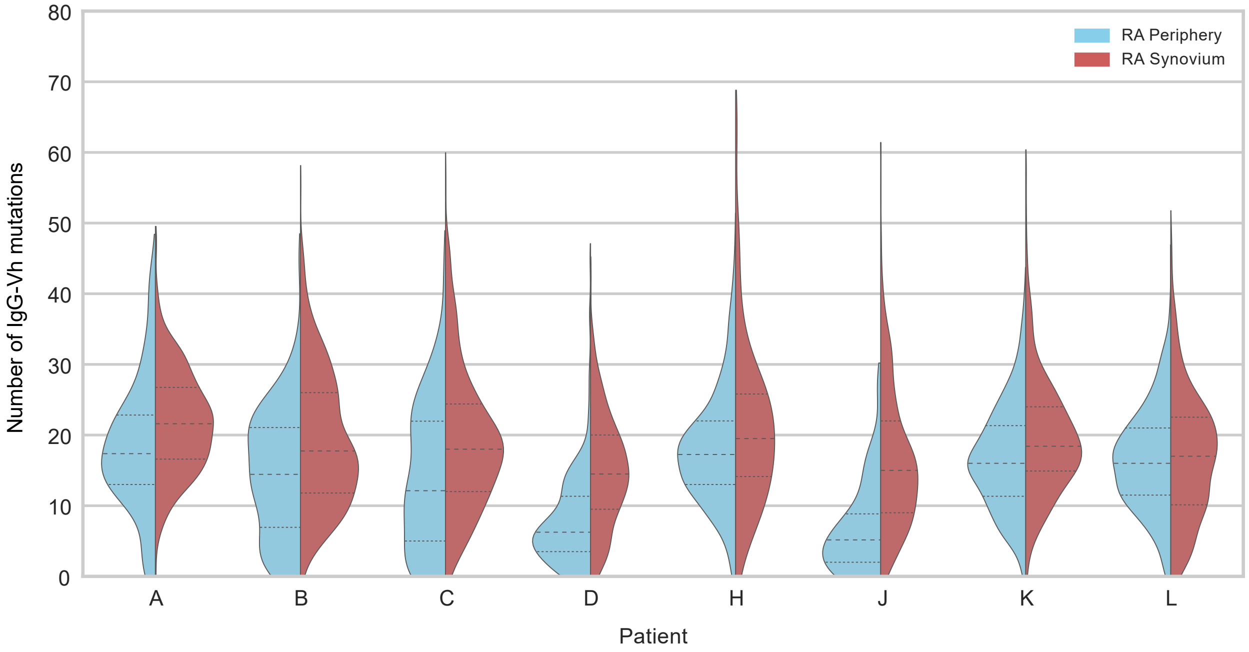

Supplementary Figure 10: Analysis of the distribution of number of mutations per clonal sequence cluster. In this analysis, clonal clusters were generated by single-linkage clustering of sequences within a CDR3 sequence Hamming distance of 1, where sequences shared the same predicted IGHV allele, IGHJ allele and junction length. To allow for variation in the number of hypermutations that may be present in a clonal cluster, the mean number of IgG-Vh mutations for all reads in the cluster is plotted.

Supplementary Figure 11

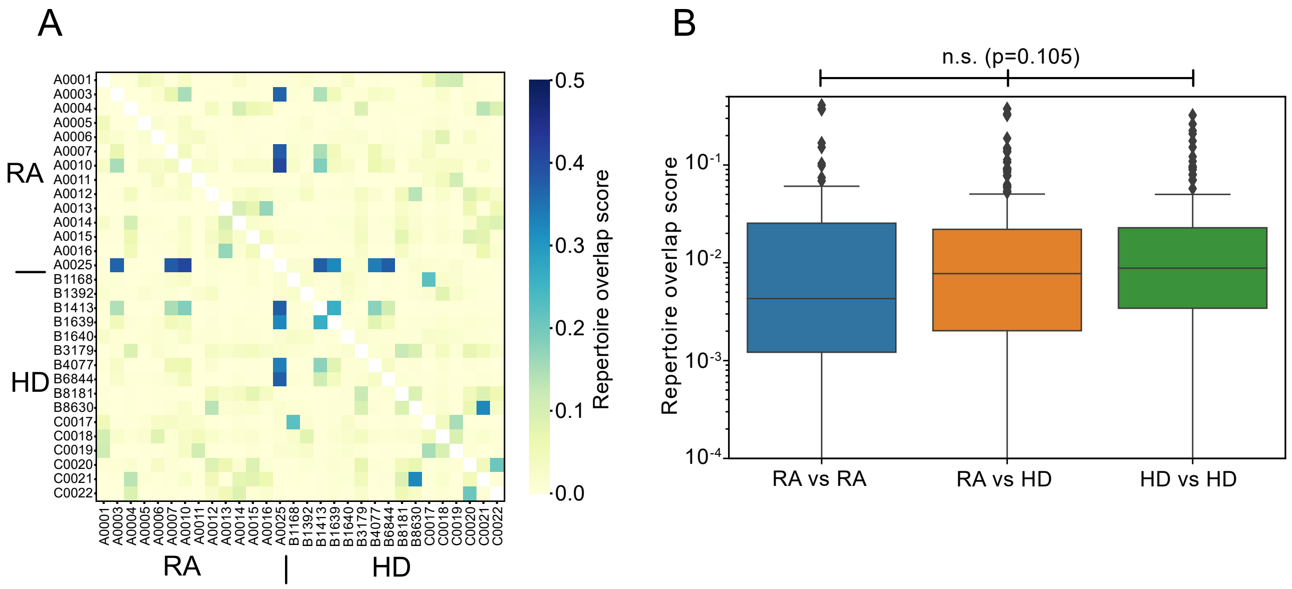

Supplementary Figure 11: Repertoire overlap analysis of RA patients and healthy donors from cohort 1 (n=14 and n=16 respectively). Clonal clusters were generated by single-linkage clustering with a Hamming distance of 1 between CDR3 sequences, where sequences shared the same predicted IGHV allele, IGHJ allele and junction length. The similarity each pair of samples was assessed using the BCR repertoire clonal overlap score, which was defined as the total number of reads from shared clonal clusters divided by the sum of sequencing reads in both samples, ranging from 0 to 1. Panel A shows a heat map of repertoire overlap score for all pairwise comparisons between RA donors and healthy donors, excluding comparisons of each donor with self. Panel B shows the repertoire overlap values for every pairwise comparison between pairs of RA donors (RA vs RA), between RA and healthy donors (RA vs HD) and between pairs of healthy donors (HD vs HD). Self comparisons are excluded. Kruskal-Wallis H test was used to test the hypothesis that the population median of all of the groups are equal (p=0.105).
